# Rational Design of MOF-Based Materials for Next-Generation Rechargeable Batteries

**DOI:** 10.1007/s40820-021-00726-z

**Published:** 2021-10-06

**Authors:** Zhengqing Ye, Ying Jiang, Li Li, Feng Wu, Renjie Chen

**Affiliations:** 1 Beijing Key Laboratory of Environmental Science and Engineering, School of Materials Science and Engineering, Beijing Institute of Technology, Beijing, 100081 People’s Republic of China; 2 Collaborative Innovation Center of Electric Vehicles in Beijing, Beijing, 100081 People’s Republic of China; 3 Advanced Technology Research Institute, Beijing Institute of Technology, Jinan, 250300 People’s Republic of China

**Keywords:** Metal–organic frameworks, MOF composites, MOF derivatives, MOF composite derivatives, Batteries

## Abstract

Metal–organic framework (MOF)-based materials with high porosity, tunable compositions, diverse structures, and versatile functionalities provide great scope for next-generation rechargeable battery applications. Herein, this review summarizes recent advances in pristine MOFs, MOF composites, MOF derivatives, and MOF composite derivatives for high-performance sodium-ion batteries, potassium-ion batteries, Zn-ion batteries, lithium–sulfur batteries, lithium–oxygen batteries, and Zn–air batteries in which the unique roles of MOFs as electrodes, separators, and even electrolyte are highlighted. Furthermore, through the discussion of MOF-based materials in each battery system, the key principles for controllable synthesis of diverse MOF-based materials and electrochemical performance improvement mechanisms are discussed in detail. Finally, the major challenges and perspectives of MOFs are also proposed for next-generation battery applications.

## Highlights

- This review summarizes recent progresses in pristine metal–organic frameworks (MOFs), MOF composites, and their derivatives for next-generation rechargeable batteries including lithium–sulfur batteries, lithium–oxygen batteries, sodium-ion batteries, potassium-ion batteries, Zn-ion batteries, and Zn–air batteries.
- The design strategies for MOF-based materials as the electrode, separator, and electrolyte are outlined and discussed.
- The challenges and development strategies and of MOF-related materials for battery applications are highlighted.

## Graphical abstract

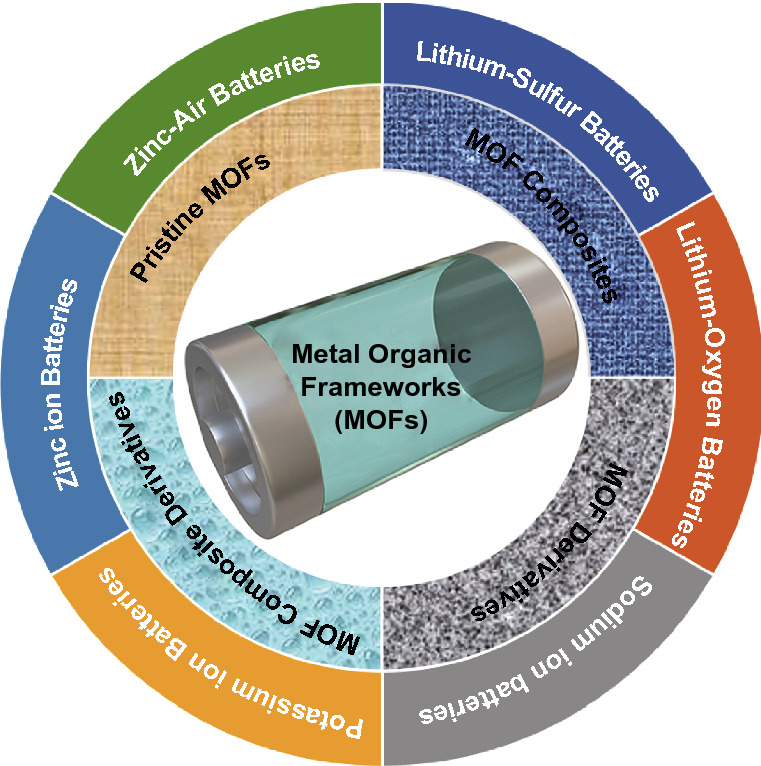

## Introduction

Over the past few decades, social attention in renewable energy storage systems has been rapidly increasing due to resource shortage and environmental degradation [1]. As one type of conventional energy storage technology, lithium-ion batteries (LIBs) with high energy density are widely utilized in mobile phones, laptops, and portable electronics [2]. However, traditional LIBs constructed with a lithiated transition metal oxide (e.g., LiCoO_2_ and LiFePO_4_) as cathode and a graphite anode are reaching their specific energy density limits [3]. Besides, LIBs are still expensive to scale up owing to the limited Li reserves. The electric vehicles (EVs) and grid-based energy-storage markets demand a high energy density and a low cost at the rechargeable batteries [4, 5]. Therefore, it is highly desirable to develop the next-generation batteries with high energy and low cost.

Lithium–sulfur (Li–S) batteries have received considerable attention because of their high theoretical specific capacity (1,675 mAh g^−1^), energy density (2,600 Wh kg^−1^), and the use of nontoxic sulfur with natural abundance and low cost [5, 6]. However, several major challenges hinder the commercialization including: (1) the depressed polysulfides conversion and low sulfur utilization resulting from the nonconductive nature of sulfur and discharged solid products (Li_2_S_2_ and Li_2_S), (2) the severe capacity fading and low Coulombic efficiency (CE) caused by the shuttle effect of soluble polysulfides, (3) the pulverization of the electrode structure originated from the volume variation (80%), and (4) the safety issue of Li dendrite formation during the charge/discharge process [7, 8]. Lithium–oxygen (Li–O_2_) batteries work broadly on the similar principle as the Li–S batteries with the only difference being in the redox reaction between Li metal anode and O_2_ cathode. Despite high energy density (3500 Wh kg^−1^) and environmentally friendly nature, Li–O_2_ batteries still face several issues. Firstly, the insulating and insoluble discharge product (Li_2_O_2_) leads to sluggish kinetics of the oxygen reduction reaction (ORR) and oxygen evolution reaction (OER). Moreover, the undesirable generation of side products (e.g., Li_2_CO_3_ and LiOH) results in inferior round trip efficiency and cycling performance [4, 9].

Advanced sodium-ion batteries (SIBs) and potassium-ion batteries (PIBs) have been regarded as one of the most promising candidates for grid-scale energy storage systems, due to the widespread distribution and low cost of sodium and potassium resources [10]. Unfortunately, the redox potential of Na/Na^+^ ( − 2.71 V vs. standard hydrogen electrode) is higher than that of Li/Li^+^ ( − 3.04 V) places, resulting in lower discharge voltage plateau and energy density of batteries [11]. Compared with Na/Na^+^, K/K^+^ possesses a lower standard redox potential ( − 2.93 V), which ensures PIBs with higher energy density. However, the large ion radius of the K^+^ (1.38 Å) leads to slow diffusion kinetics, thus resulting in low capacity, inferior rate performance, and poor cycling stability during the insertion/deinsertion process [12]. Aqueous zinc (Zn) batteries (such as Zn-ion batteries and Zn–air batteries) have recently drawn considerable interest owing to their high theoretical capacity (820 mAh g^‒1^), low toxicity, high safety, and low cost [13, 14]. However, they also suffer from several issues that need to be addressed. The development of aqueous Zn-ion batteries (ZIBs) is plagued by the scarcity of suitable cathode materials for Zn-ion storage. For Zn–air batteries, the sluggish oxygen electrocatalytic kinetics usually cause a large overpotential and poor cycling stability. Moreover, Zn batteries face the problem of dendrite formation in anode resulted from uneven charge distribution and side reactions during plating/stripping. Dendrite growth is responsible for poor cycle life, capacity fade, and safety problems for aqueous Zn batteries [15].

To address the aforementioned challenges in each next-generation battery system, a large number of works have been devoted to exploring new materials with high electrochemical performance. Metal–organic frameworks (MOFs), constructed from metal ions or clusters and organic ligands, have attracted tremendous interest as a new class of porous materials in various fields, such as drug delivery [16, 17], gas adsorption, and separation [18, 19], energy storage and conversion [20, 21]. MOFs possess topologically diverse and well-defined structures, resulting from their underlying topological nets. The combination of metal nodes (ions or clusters) and organic linkers endows MOFs’ diverse structures with abundant elemental compositions and tunable porosity. The unique pore structure of MOFs ensures rapid electrolyte penetration and ion diffusion. The controllable structures and designable components could not only enable prominent electrochemical stability from their robust structure, but also guarantee a high capacity with their abundant electroactive sites. However, MOFs are rarely utilized directly as electrode materials for batteries due to their poor electrical conductivity [22]. Comparatively, MOF composites and MOF-derived materials not only inherit the structure, porosity, and composition advantages of MOFs, but also achieve improved electrical conductivity offered by the functional components. As a result, the electrochemical performances of the MOFs/MOF composites and their derivatives can be further improved, which opens up a new avenue for the rational design of battery materials for energy storage.

The present article focuses on the recent progress in pristine MOFs, MOF composites, MOF derivatives, and MOF composite derivatives for next-generation rechargeable batteries (SIBs, PIBs, ZIBs, Li–S, Li–O_2_, and Zn–air batteries). We comprehensively discuss the unique advantages of components, structures, and properties in electrode materials, separators, electrolytes, and metal anodes for improved battery performance. The key factors for controllable preparation of various MOF-related materials and battery performance enhancement mechanisms are presented in detail. The main challenges and prospective solutions of these design strategies are proposed. We hope that this review will help guide and inspire the future design of advanced MOF-based materials for next-generation rechargeable batteries.

## Classification and Characteristics of MOF-based Materials

### Classification of MOF-Based Materials

**Fig. 1:**
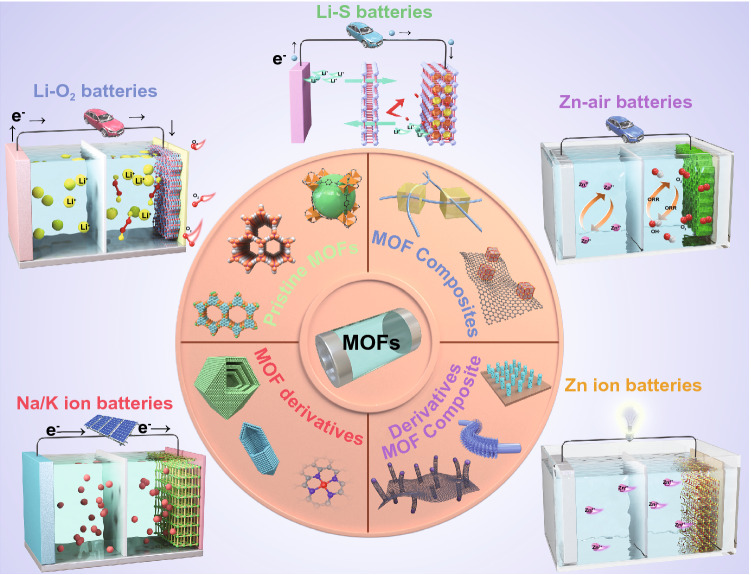
Schematic illustration of MOF-related materials for next-generation batteries

Generally, pristine MOFs consisted of metal ions or clusters and organic ligands by coordination bonds. MOFs possess unique compositional and structural superiorities compared with conventional materials. In terms of component advantages, various metal nodes and organic linkers can be utilized to synthesize MOFs with different physical and chemical properties. In addition, pristine MOFs possess diverse nanoarchitectures such as 0D nanoparticles, 1D nanotubes, 2D nanosheets, and 3D nanoarrays with high porosity, which can provide high exposure of active sites and fast mass transport for high-performance batteries. Furthermore, MOFs can be incorporated with functional materials such as carbon, polymers, metal nanoparticles, and functional molecules to construct MOF composites. The synergistic effects between MOFs and the functional components can contribute to the enhanced electrochemical performance of the MOF composites. Besides, the transformation of MOFs into diverse MOF derivatives (e.g., carbon materials, metal/metal compounds, and single-atom sites) by post-synthetic strategies can result in novel properties over pristine MOFs. Similarly, MOF composite-derived (e.g., MOF/carbon, MOF/polymer, and MOF/metal compounds) multifunctional superstructures can further enrich their structural diversities, which are beneficial for the improved overall performance of batteries. Based on the above discussion, the MOF-based materials have been classified into four main groups: pristine MOFs, MOF composites, MOF derivatives, and MOF composite derivatives (Fig. 1). Furthermore, these MOF-related materials show great potential in many battery applications, including SIBs, PIBs, ZIBs, Li–S, Li–O_2_, and Zn–air batteries.

### Characteristics of MOF-Based Materials

MOFs with tunable chemical compositions and crystalline porous structures display the following unique characteristics: (1) crystalline porous frameworks. Porosity is an intrinsic feature of MOFs because of the formation of open frameworks by coordination between metal nodes and organic linkers. Moreover, the original porous frameworks can also be converted into hierarchically porous structures after post-synthetic treatment. The unique porous frameworks ensure uniform distribution of the internal active sites, which is favorable to the continuous proceedings of electrochemical reaction. The encapsulation of other active species in the porous frameworks can also prevent the formation of severe agglomerates, thus resulting in enhanced electrochemical performance. (2) Tunable metal nodes. Metal nodes play a vital role in determining the porous frameworks and functional properties of MOFs [23]. For example, the MOFs with bimetallic active sites can also be synthesized via manipulating metal nodes, which can significantly facilitate electron transport and electron distribution, contributing to enhanced electrical conductivities and stabilities. Consideration of the atomic isolation of metal nodes is an intrinsic characteristic of MOFs, MOF-derived single-atom catalysts (SACs) with atomically dispersed metal active sites, which maximizes the catalytic activity of metal nodes. (3) Diverse organic ligands. Organic ligands can serve as “skeletons” in MOFs and isolate the metal nodes from each other, resulting in uniformly distributed metal active sites. Furthermore, rich functional groups in organic ligands promote the heterogeneous nucleation and uniform growth of MOFs on other functional materials through weak interactions in solutions [24]. For MOF derivatives, diverse organic ligands are not only the source of carbon, but also the source of other nonmetallic dopants, such as nitrogen, sulfur, and phosphorus [25]. Active dopants in the MOF derivatives further improve the electrochemical performance because of local electron transfer and redistribution. In addition, some nonmetal elements could also react with metal cations, partially converting metals into metal sulfides, metal phosphides, or metal–N_x_ active species, which is beneficial for enhanced battery performance [26, 27]. These characteristics of MOFs demonstrate unique advantages when compared with other conventional materials, which is vital to achieving high-performance next-generation batteries.

## Lithium–Sulfur Batteries

### Pristine MOFs

**Fig. 2:**
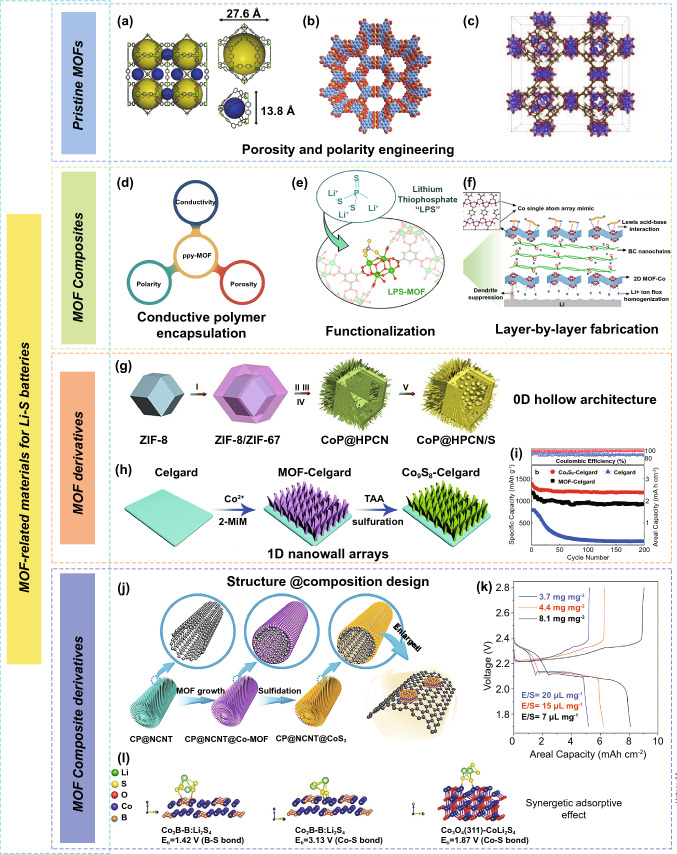
MOF-related materials for Li–S batteries. **a** Structure of Ni-MOF (yellow and blue spheres represent pore volume; gray, C; red, O; blue, N; green, Ni) [29]. Copyright © 2014, American Chemical Society. **b** Crystal structure of Ni_3_(HITP)_2_ [30]. Copyright © 2018, Wiley–VCH. **c** crystal structure of MOF-199 [33]. Copyright © 2019, Elsevier. **d** Three criteria of ppy-MOF structure [22]. Copyright © 2018, Wiley–VCH. **e** Scheme of Li_3_PS_4_-functionalized Zr-MOFs host with encapsulation of polysulfides [44]. Copyright © 2019, American Chemical Society. **f** Schematic representation for Li–S batteries of the B/2D MOF-Co separator [47]. Copyright © 2020, Wiley–VCH. **g** Schematic illustration of synthesis process for CoP@HPCN/S [56]. Copyright © 2019, Elsevier. **h** Schematic illustration of Co_9_S_8_-Celgard preparation and **i** cyclic stability of the cells with the various separators [59]. Copyright © 2018, The Royal Society of Chemistry. **j** Schematic illustration of CP@NCNT@CoS_3_ synthesis [69]. Copyright © 2019, Wiley–VCH. **k** Charge–discharge profiles of CC@CS@HPP sulfur electrodes at 0.01 C [70]. Copyright © 2020, Wiley–VCH. **l** First-principles calculations of the chemical interaction of Co_2_B and Co_3_O_4_ surfaces with polysulfides [78]. Copyright © 2019, American Chemical Society

Pristine MOFs with an abundant pore structure, well-defined morphology, and high polarity possess advantages in terms of accommodating active sulfur, alleviating volume variation, inhibiting polysulfides shuttling behavior, and protecting Li metal anode. The pioneering work of mesoporous MIL-100 (Cr) (MIL, Matérial Institut Lavoisier) as a sulfur host for Li–S batteries in 2011 was reported by Tarascon and co-workers [28]. Although the successful incorporation of 48 wt% sulfur into the MIL-100 (Cr) with a high surface area, the battery delivered poor cycle stability due to the weak binding between the polysulfides and oxygenated MOF groups. In 2014, Xiao et al. [29] proposed interwoven microporous and mesoporous Ni-MOF (Ni_6_(BTB)_4_(BP)_3_ (BTB = benzene-1,3,5-tribenzoate and BP = 4,4’ bipyridyl)) in which Ni(II) served as a Lewis acid, while polysulfides acted as a Lewis base (Fig. 2a). The Ni-MOF can strongly confine polysulfides within the cathode side via physical and chemical interactions, leading to the excellent cycling stability (the capacity retention of 89% after 100 cycles at 0.1 C) of Ni-MOF sulfur cathode. However, the insulating nature of MOFs leads to poor sulfur utilization, and the framework could suffer from gradual degradation, especially after long-term cycling.

MOFs with large surface area and tunable porosity could be appropriate candidates as ionic sieves to suppress these shuttling polysulfide ions. For example, Fang and co-workers [30] demonstrated that conductive and microporous MOF with the polysulfide-capturing ability (Ni_3_(HITP)_2_ (HITP = 2,3,6,7,10,11-hexaiminotriphenylene)) was grown in situ on the separator for decreasing the polysulfide shuttling behavior (Fig. 2b). The Li–S batteries with MOFs’ functional separator achieved a high area capacity of 7.24 mAh cm^−2^ (capacity retention of 86%) after 200 cycles at 0.5 C under high sulfur loading of 8.0 mg cm^−2^. Subsequently, a simple wet-chemistry method was developed for the preparation of ultrathin MOF (Cu_2_(CuTCPP)) nanosheets [31]. The as-obtained MOF nanosheets were used to assemble a highly oriented microporous membrane modified separator for inhibiting the polysulfide shuttling, which significantly enhanced cycling stabilities.

MOFs with narrow and uniform pore size can not only suppress the polysulfides shuttle in Li–S batteries, but also help to protect the Li metal anode for inhibiting the growth of Li dendrites. Zhou and co-workers designed MOF-based membrane by using copper benzene tricarboxylate (HKUST-1) nanoparticles as assemble units and the PVDF-HFP as a binder [32]. The highly homogenous pore sizes of MOF particles boost uniform Li^+^ fluxes, fundamentally suppressing the Li dendrites growth and leading to stable Li plating/striping even at a high current density of 10 mA cm^−2^. The Li–S batteries with a MOF-based separator displayed an ultralong cycle life with a low-capacity decay rate of 0.015% per cycle after 2000 cycles. Recently, Chen’s group has demonstrated that MOF-199 could inhibit the growth of Li dendrites by serving as a robust shield and homogenize Li^+^ concentration by their abundant porous structure (Fig. 2c) [33]. However, the intrinsic mechanical brittleness of the MOFs made them hardly meet the practical requirements of durable and stable Li–S batteries.

### MOF Composites

Despite the attractive advantages, the application of the MOFs materials is hindered by several challenges, including the poor conductivity and detrimental electrochemical stability of MOFs [34, 35]. The combination of MOFs with various functional materials is a facile and effective way to further enhance MOF-based Li–S battery performance. Numerous MOF/carbon composites have been presented by assembling MOFs, highly conductive and excellent mechanical carbon species, including carbon nanotubes (CNTs) and graphene, and act as sulfur host and separator for improved Li–S battery performance [35–38]. For example, the cerium (Ce)-MOF-2/CNT composites were fabricated as a modified separator material for high-performance Li–S batteries [39]. The excellent performance is ascribed to the synergistic effect of strong adsorption and catalytic transformation of the Ce-MOF-2 toward polysulfides along with the highly conductive and robust CNT. In this regard, Liu et al. [40] synthesized a range of MOFs on graphene nanosheets including 2D Ni-2,6-NDC (2,6-NDC = 2,6-naphthalene dicarboxylate), zeolitic imidazolate framework-8 (ZIF-8), HKUST-1, and hybrid NiFe-BTC (BTC = benzene-1,3,5 tricarboxylate). Utilization of ZIF-67/graphene nanosheet-based membranes in Li − S batteries results in higher long-term cycling stability compared to bare graphene and granular ZIF-67 + G membranes. Moreover, the construction of designed composites of MOFs with conductive polymers seems to be an effective strategy for the realization of excellent sulfur electrode materials [41]. For instance, a conductive polypyrrole (PPy) was applied to construct PPy-PCN-224 composite for a long-term cycle at a high rate (specific capacity of 780 mAh g^−1^ after 400 cycles at 5 C) in Li–S batteries (Fig. 2d) [22]. This method combines the polarity and cross-linked pore and tunnels of PCN-224 with the conductive gain of PPy; thus, their geometric advantages could be fully utilized. A substantially higher conductivity additive can be added to enhance the MOF conductivity, but resulting in a lower energy density in Li–S batteries.

Functionalization strategy can be also used to design a variety of MOF composites [42, 43]. For example, Thoi and co-workers [44] proposed lithium thiophosphate (Li_3_PS_4_)-functionalized zirconium (Zr)-MOFs with sulfur as cathode with enhanced rate capabilities (Fig. 2e). The incorporation of Li_3_PS_4_ in Zr-MOFs enhances sulfur utilization and polysulfide confinement to maintain a high reversible capacity over prolonged cycling. The inorganic Li_3_PS_4_ with excellent stability and large surface area of MOFs also prevents battery damage under high rates and poor cycling conditions. The decorated MOF channels (pore size of 9.0 Å, Ms-9.0) with negatively charged sulfonic polymer (NSP) were reported as a modified separator for enhanced cycling stability of Li–S batteries [45]. The Ms-9.0-NSP separator can form sulfurphobic interaction between NSP and polysulfides, which could facilitate the Li^+^ diffusion, reduce voltage polarizations, and relieve initial “sulfur loss.”

An effective strategy to construct composite structures with MOFs and polymers assures both inhibiting shuttling effect and suppressing Li dendrite growth in Li–S batteries simultaneously [46]. A layer-by-lawyer (LBL)-assembled bifunctional separator (B/2D MOF-Co) was prepared by employing bacterial cellulose (BC) and ultrathin MOF-Co nanosheets [47]. The Co single-atom array mimic on 2D ultrathin MOF can not only homogenize Li^+^ flux via strong Li^+^ adsorption with O atoms, but also effectively trap polysulfides through Lewis acid–base interaction (Fig. 2f). Consequently, the B/2D MOF-Co can simultaneously regulate the Li stripping and plating behavior and migration of polysulfides, thus achieving the safety and life of Li–S batteries. Recently, Gao et al. [48] fabricated a MOF-based triple-layer kind of separator with stepped channels through the combination of multidimensional various MOFs and functional polymers. This MOF/polymer triple-layer separator with stepped channels can inhibit polysulfides shuttling, promote the efficient transfer of Li^+^/electrolyte, and suppress Li–S battery polarization. As a powerful separator, it displays superior cycling performance compared to single-layer and double-layer membranes. Besides, a MOF-modified gel polymer electrolyte (GPE) was constituted of Mg(II)-based MOF material (Mg-MOF-74) and poly(vinylidene fluoride) (PVDF) polymer for high-performance quasi-solid-state Li–S batteries [49]. Because of the unique pore structure, the Mg-MOF-74 material can not only inhibit the soluble polysulfides diffusion but also cage TFSI^−^ anions, thus boosting a uniform flux of Li^+^ and a stable Li metal anode.

### MOF Derivatives

In addition to MOF composites, MOFs can be directly transformed into nanostructured porous carbon, metal compounds, and their composites. These MOF derivatives possess hierarchically porous structures, excellent conductivity, and abundant polar/catalytic sites that are beneficial for enhanced Li–S battery performance [50–52]. Specifically, the porous carbon substrate can efficiently promote ion/electron transport and physically confine polysulfides, while elaborately designed hollow structures and core–shell structures can relieve volume expansion and preserve structural integrity during cycling [53, 54]. Furthermore, the incorporated metal compounds can offer polar/catalytic sites to chemically immobilize polysulfides and efficiently catalyze the sulfur species conversion reaction [55]. For example, Ye et al. [56] reported the hollow polyhedra/CNT confined CoP nanoparticles superstructures (CoP@HPCN) derived from core–shell ZIF-8/ZIF-67 as a sulfur host (Fig. 2g). It was proposed that smart hollow polyhedra/CNT architecture for alleviating volume variation and boosting ion/electron transport, together with the adsorption and catalysis effect of CoP nanoparticles for polysulfides transformation, contributed to an outstanding electrochemical performance in Li–S batteries.

MOF derivatives of modified separators can suppress polysulfides shuttle via chemical interaction and rapid redox kinetics [57, 58]. The representative study of 2D ZIF-67-derived Co_9_S_8_-Celgard separator was proposed by Manthiram and co-workers [59]. The 2D ZIF-67 was in situ grown on the Celgard and then was chemically converted into Co_9_S_8_-Celgard via solvothermal sulfurization (Fig. 2h). The well-aligned Co_9_S_8_ hollow nanowall arrays as a multifunctional polar barrier enabled a high capacity of 1385 mAh g^−1^ with a capacity retention of 86% after 200 cycles for Li–S batteries (Fig. 2i). In another case, Ni/Zn-bio-MOF-100-derived bimetal carbide Ni_3_ZnC_0.7_ possesses both sulfophilic sites of Ni and lithiophilic sites of Zn, resulting in strong adsorption toward polysulfides and reduced energy barriers for Li^+^ diffusion [60]. When acting as the separator coating, the Ni_3_ZnC_0.7_ could effectively suppress the shuttle effect of polysulfides, leading to the excellent performance of Li–S batteries even at a high rate of 7 C and high sulfur loading (6.8 mg cm^−2^). In addition, the use of MOF derivatives as separators to simultaneously suppress Li dendrite growth and inhibit polysulfides shuttle behavior has also been reported recently. The amorphous TiO_2_ embedded in benzene-1,4-dicarboxylic acid (denoted as a-TiO_2_-BDC) derived from Ti-containing MOF (MIL-125-Ti) was coated on a commercial separator to stabilize Li metal anodes for Li–S batteries [61]. The a-TiO_2_-BDC not only induces the formation of robust solid electrolyte interphase (SEI) layer, but also strongly adsorbs polysulfides, which give an advantage to the anode and cathode of Li–S batteries. However, the poor compatibility between the MOF derivatives and the separators could be unable to support the practical batteries for long-term cycling.

### MOF Composite Derivatives

MOF composite derivatives have gained much attention owing to their applications in Li–S batteries [62–65]. Combining MOF derivatives with conductive matrix (e.g., graphene, CNT, and carbon cloth) is a promising strategy to achieve an excellent electrochemical performance because of the reduced mechanical stress and prevented self-aggregation [66–68]. For instance, Sun and co-workers proposed [69] the use of amorphous CoS_3_ as an electrocatalyst to promote the transformation of Li_2_S_2_ to Li_2_S. First, the nitrogen-doped carbon nanotubes were grown on carbon paper (CP@NCNT) as catalyst support (Fig. 2j). Then, Co-MOF was grown on the surface of CP@NCNT and further transformed into the CP@NCNT@CoS_3_. The Li_2_S/Li_2_S_2_ ratio in the discharge products increased to 5.60/1 from 1/1.63 with CP@NCNT@CoS_3_ via XPS analysis, contributing to 80% sulfur utilization and the high-capacity retention during cycling under high-sulfur-loading conditions. Recently, Ye et al. [70] constructed a high-efficiency CoSe electrocatalyst with hierarchical porous polyhedron on a carbon cloth framework (CC@CS@HPP) through simply immersing carbon cloth in the ZIF-67 precursors and followed by in situ selenization strategy. A freestanding CC@CS@HPP significantly accelerated polysulfide capture/diffusion and Li_2_S precipitation/decomposition, which achieved a high areal capacity of 8.1 mAh cm^−2^ at high sulfur loading of 8.1 mg cm^−2^ under a lean electrolyte (Fig. 2k).

Developing fabrication strategies of metal compounds/MOF composite derivatives is also of great importance [71–73]. By compositing with proper metal compounds, MOF composite derivatives could offer more exposed active sites for polysulfides regulation and tailorable structures for sulfur loading, thus improving the sulfur utilization and enhancing sulfur loading simultaneously. For example, the TiO_2_ and Co nanoparticle-decorated carbon polyhedra (C–Co/TiO_2_) were prepared via titanium tetraisopropanolate­containing ZIF­67 as precursor through pyrolysis treatment as described in the previous study [74]. When serving as cathode materials for Li–S batteries, C–Co/TiO_2_ polyhedras show significantly improved electrochemical performances due to high-efficiency conductive networks, robust architecture, abundant TiO_2_- and Co-adsorption sites. In another study, Chen and co-workers reported a hollow nanocage-like layered double hydroxides/Co_9_S_8_ (H-LDH/Co_9_S_8_) heterostructure by in situ construction and sulfurization of ZIF-67 templated NiCo-LDH [75]. The robust H-LDH/Co_9_S_8_ sulfur host could inhibit the polysulfides diffusion and accommodate sufficient sulfur owing to abundant O-containing groups and Co–S sites. Moreover, the intimated interfaces of NiCo-LDH shell and Co_9_S_8_ domains present greatly enhanced electron conductivity and Li^+^ diffusivity.

Applying MOF composite derivatives as separators for Li–S batteries has the potential to block polysulfides [76, 77]. For example, Co_2_B@CNT was prepared as a functional separator by employing ZIF-67 and CNT [78]. Interestingly, both Co and B in Co_2_B could bond with the S_4_^2−^ anions and therefore exhibit higher adsorption capability when compared with Co_3_O_4_ (Fig. 2l). By combining the synergetic adsorptive effect of Co_2_B and the electron highway of CNT, the cell with modified separators exhibited prominent cycling life with a capacity decay rate of 0.0072% per cycle after 3000 cycles and ultrahigh-rate capability (1172.8 mAh g^−1^ at 5 C). Also, MOF composite derivatives can serve as two-in-one hosts for both sulfur cathode and metallic Li anode to improve their performance simultaneously [79]. As an example, the use of bimetallic Co/Zn-ZIF and graphene nanosheet substrate was proposed as precursor for the synthesis of superhierarchical Co-embedded N-doped porous carbon nanosheets (Co/N-PCNSs) [80]. The Co nanoparticles and doped N heteroatoms can work synergistically to confine soluble polysulfides and boost the conversion kinetics of sulfur cathode. Meanwhile, the hierarchical porous structure and the lithiophilic N heteroatoms in Co/N-PCNSs can regulate Li nucleation and inhibit Li dendrite growth in the anode. As a result, a full Li–S battery with Co/N-PCNSs as two-in-one hosts achieves excellent capacity retention and stable CE.

### Summary

Li–S batteries as prominent candidates of next-generation batteries have been considered as rapid development. Because of the multielectron reaction mechanism, many issues still exist in Li–S batteries, including the low-sulfur utilization, sluggish sulfur conversion, polysulfides shuttle, and Li dendrite growth. As great as single MOFs, MOF composites and their derivatives perform in Li–S batteries. However, the poor conductivity of MOFs, the self-agglomeration and low tap density of MOF derivatives, the lack of diversity of the MOF composites, and their derivatives are worth to be comprehensive consideration. The emerging MOF composites ion sieve and 3D MOF composite derivatives for freestanding sulfur cathodes are very likely to be good choices for high-performance Li–S batteries.

## Lithium–Oxygen Batteries

### Pristine MOFs

**Fig. 3:**
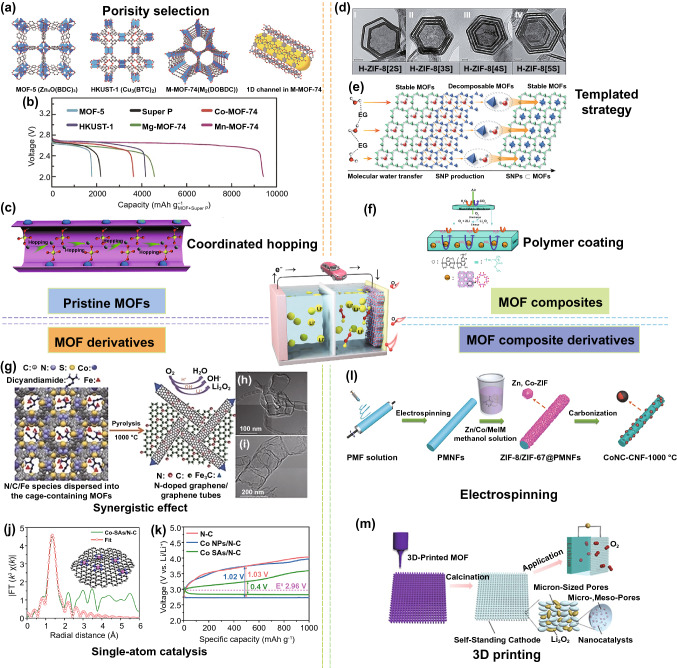
MOF-based materials for Li–O_2_ batteries. **a** Crystal structures and **b** discharge profiles of pristine MOFs [81]. Copyright © 2014, Wiley–VCH. **c** Schematic illustration of Li diffusion path in pore channel of Cu-MOF-74. (O atom: red, Cl atom: yellow, Cu atom: blue) [83]. Copyright © 2019, The Royal Society of Chemistry. **d** TEM images of H-ZIF-8[2S, 3S, 4S, 5S]. All scale bars: 50 nm. **e** Schematic illustration of the formation process of SNP-embedded H-ZIF-8[nS] (green: Zn, violet: Co) [85]. Copyright © 2020, Wiley–VCH. **f** Schematic illustration of the MMM based on MOF composites for rebelling H_2_O and CO_2_ molecules in Li–O_2_ batteries (Al, pink; O, red; C, gray; H, blue) [86]. Copyright © 2015, Royal Society of Chemistry. **g** Schematic illustration and **h, i** TEM images of N-doped graphene/graphene-tube [88]. Copyright © 2014, Wiley–VCH. **j** XAFS measurements and **k** discharge–charge curves of Co-SAs/N–C. Reproduced with permission. [95] Copyright 2020, Nature Publishing Group. **l** Synthesis process for CoNC-CNFs [97]. Copyright © 2018, Wiley–VCH. **m** Schematic illustration of 3DP-NC-Co O_2_ cathode for Li–O_2_ batteries [98]. Copyright© 2019, Wiley–VCH

MOFs are a viable option for Li–O_2_ batteries owing to the tunable pore structure, accessible metal sites, and robust framework structure. Wu et al. [81] demonstrated that MOFs with accessible metal sites could contribute to a significant O_2_ enrichment/diffusion in the framework (Fig. 3a). Five MOFs (MOF-5, HKUST-1, Co-MOF-74, Mn-MOF-74, and Mg-MOF-74) were studied, among which Mn-MOF-74 with 1D regular channels and open metal sites delivered the highest discharge capacity of 9420 mAh g^−1^ at 50 mA g^−1^, which was more than four times for MOF-free cathode (Fig. 3b). Moreover, robust Mn-MOF-74 exhibited excellent structural stability without obvious decomposition after discharge/charge. A bimetallic MOF (MnCo-MOF-74) was presented to further enhance the performance of Li–O_2_ batteries [82]. Benefitting from both Mn–metal and Co–metal clusters, MnCo-MOF-74 enhanced reversibility and efficiency during repeated cycles.

Recently, a single-ion conductor in the liquid electrolyte was prepared by coordinating the anions in the electrolyte on the abundant metal sites of a Cu-MOF-74 rod-like substrate [83]. Further investigations demonstrate that the Li^+^ migrate within the pores of the Cu-MOF-74 by a Grotthuss-like mechanism that the charge is transferred by coordinated hopping of solvated Li^+^ between the lithiophilic ClO^4–^ groups (Fig. 3c). The single-ion electrolyte can contribute to homogeneous single Li^+^ transport in the electrolyte and effectively suppress Li dendrites growth. When the single ion electrolyte is applied for Li–O_2_ batteries, an enhanced cycle performance with low overpotential is achieved.

### MOF Composites

Considering most MOFs possess low electrical conductivity, it is desired to develop strategies to improve the conductivity of MOFs, such as hybridization with conductive matrix and functionalization with guest molecules. Mn-MOF-74 nanoparticles were directly grown on 1D CNTs by a simple additive-mediated synthesis as cathode materials for Li–O_2_ batteries [84]. The Mn-MOF-74@CNTs could provide conductive networks and prevent the agglomeration of MOF nanoparticles, thus exhibiting fewer side reactions and improved cycling performance in a humid oxygen environment. Recently, dinuclear Co(OH)_2_ sub-nanometric particles (SNP) within multishell hollow ZIF-8 (H-ZIF-8[nS], where n is the number of shells) were autogenously synthesized by Kang and co-workers (Fig. 3d) [85]. First, the stable MOF (ZIF-8) layers effectively transferred ethylene glycol (EG) isolated water molecules to the decomposable MOF (ZIF-67) layers through hydrophobic micropores. Subsequently, SNPs derived from the decomposable ZIF-67 were stabilized inside the pore channels of H-ZIF-8 (Fig. 3e). The hopping charge transport between SNPs stabilized by π-back bonding introduces high electrical conduction in MOFs, thus leading to high capacities and low overpotentials in Li–O_2_ batteries.

For more practical lithium–air batteries (LABs), MOF composites could be applied as an O_2_-permeable membrane to protect cathodes from moisture and CO_2_ in ambient air atmosphere. In one example, a mixed matrix membrane (MMM) was constructed by introducing a polydopamine-coated AI-based MOF (CAU-1-NH_2_) into a polymethylmethacrylate (PMMA) substrate (Fig. 3f) [86]. The abundant -NH_2_ groups in CAU-1-NH_2_ can efficiently capture CO_2_ while the PMMA endowed the MMM with excellent hydrophobic nature. Therefore, the LABs with MMM achieved high discharge capacity and excellent cycling stability under a real ambient atmosphere (humidity = 30%). In addition, the MOF@PVDF-HFP composite separator acts as a dual redox mediator molecule sieve to suppress the shuttling and protect Li metal for Li–O_2_ batteries [87]. The Li–O_2_ batteries achieved a superior cycled life over 100 cycles (5000 mAh g^−1^) at high current rate of 1000 mA g^−1^.

### MOF Derivatives

MOF derivatives with high electrical conductivities, hierarchical porous structure, and well-distributed catalysts are favorable to the mass transport, oxygen redox reactions, and storage of discharged products. The earliest work on MOF-derived cathode catalyst for Li–O_2_ batteries was proposed by Wu and co-workers [88]. The in situ formation of Fe- and N-doped graphene/graphene-tube nanocomposites were prepared by from the cage-containing MOF (Fig. 3g–i). The designed Co-MOF is used as a precursor to be further annealed with dicyandiamide and iron acetate to prepare a Fe- and N-doped graphene/graphene tube catalyst. The doping of pyridinic- and quaternary-N and coordination with iron (Fe-N_x_) creates more active sites for boosting the adsorption of O_2_ and the dissociation of O–O bonds. Besides this, N-doping Co@graphene [89], Co_3_O_4_-carbon [90], NiCo_2_O_4_ nanoflake arrays [91], ZnO/ZnFe_2_O_4_/carbon nanocages [92], and Co_9_S_8_@carbon porous nanocages [93] have also been reported as cathode catalyst for Li–O_2_ batteries.

Dual MOF-derived (MIL-100(Fe) and ZIF-8) Fe–Fe_3_C-embedded Fe–N-codoped carbon was proposed as an excellent ORR electrocatalyst for aprotic Li–O_2_ batteries [94]. The Fe-Fe_3_C@Fe–N–C dual active sites improve oxygen affinity and accelerate *OH desorption. The partial graphitization carbon with hierarchical porosities (micro-/macro-pores) maximized the active sites and promote mass transport. Recently, Yin’s group synthesized desirable Co single atoms embedded in 2D MOF-derived (Zn–hexamine complex) N-doped carbon substrate (Co-SAs/N–C) as a catalyst for Li–O_2_ batteries (Fig. 3j) [95]. Taking advantages from both 2D MOFs and uniformly distributed atomic Co sites, the elaborately designed Co-SAs/N–C catalyst is to help for accelerating formation/decomposition of nanosized Li_2_O_2_. As a result, the Co-SAs/N–C cathode can afford superior discharge capacity, ultra-low charge/discharge polarization (0.40 V) (Fig. 3k), and excellent cyclability (260 cycles at 400 mA g^−1^).

### MOF Composite Derivatives

The construction of MOF/conductive matrix derivatives is conducive to further improve electronic conductivity and prevent aggregation of MOFs, thus maximizing catalytic active sites for ORR and OER reaction in Li–O_2_ batteries. A dual-phasic carbon cathode for enhanced Li–O_2_ batteries was prepared by adopting ZIF8/CNT composite [96]. The dual-phasic nanoarchitecture combines the advantages of both components: MOF-derived carbon endows a high surface area for the oxygen reactions and a large pore volume for Li_2_O_2_ accommodation, and CNTs provide rapid electron/O_2_ transport pathways and additional void spaces for Li_2_O_2_ storage. Moreover, a bimetallic ZIF-8/ZIF-67 and polyacrylonitrile nanofibers composite-derived, Co-N_x_-confined porous carbon nanofibers (CNFs) with graphitic carbon-embedded Co nanoparticles (CoNC-CNFs) were prepared and used as an efficient dual catalyst for both ORR and OER (Fig. 3l) [97]. The excellent catalyst performance is attributed to the high graphitized 1D carbon structure for fast electronic mobility, hierarchical porosities for mass transport, and uniformly dispersed CoN_x_C active sites for functionalized carbon network.

In another interesting study, Wang and co-workers reported two MOF composite-derived cathodes for Li–O_2_ batteries, namely the Co-MOF/carbon paper derived Co nanoparticles assembled in N-doped porous carbon flakes (CP-NC-Co), and 3D-printing Co-MOF/Pluronic F127 derivative (3DP-NC-Co) [98]. The hierarchical porous framework of MOF composite derivatives significantly promotes the deposition of Li_2_O_2_ particles and accelerates their decomposition because of the confinement of nonconductive Li_2_O_2_ within the pores and the existence of Co electrocatalysts. Moreover, they found that 3DP–NC–Co exhibited a higher discharge capacity, a lower overpotential, and longer cycle performance than that of CP–NC–Co, which resulted from a light-weight and unique hierarchically porous framework of 3DP–NC–Co (Fig. 3m). Recently, Hu et al. synthesized Ru single atoms distributing in N-doped porous carbon on carbon cloth (Ru SAs-NC/CC) by ionic substitution and spatial confinement strategies [99]. Importantly, single atomic catalysts could maximize the redox efficiency and reversibility of Li–O_2_ batteries using the Ru–N_4_ catalytic active centers, while the carbon cloth with sufficient electronic conductivity can promote mass transfer and with enough porous channels can accommodate the discharge product Li_2_O_2_. As expected, the Ru SAs-NC/CC electrode can deliver the lowest overpotential (0.55 V at 0.02 mA cm^−2^) compared with pyrolyzed ZIF-8 and Ru nanoparticles’ counterparts.

### Summary

In summary, MOF/MOF composites and their derivatives show great potentials for Li–O_2_ batteries due to their unique pore channels, open metal active sites, and structural stability. However, many fundamental and technical challenges need to be overcome before MOF-based materials can meet the requirements of practical Li–O_2_ batteries. At the current stage of research, MOF/MOF composite derivatives show high electric conductivities and good chemical stabilities, which can be directly used as the bifunctional catalyst for Li–O_2_ batteries. A better understanding of catalytic mechanisms in Li–O_2_ chemistry is highly desirable to guide and explore more MOF-derived cathode materials. The pore structure and functional active sites of MOFs require to be optimized for application as separator and electrolyte framework in Li–O_2_ batteries. The development of MOF-based materials with high polarity, hierarchal structure, and abundant lithiophilic sites is also expected to protect Li metal anodes in Li–O_2_ batteries.

## Sodium-Ion Batteries

### Pristine MOFs

MOFs such as Prussian blue and its analogues (PB and PBAs) with open framework and interstitial sites ensuring facile insertion/extraction of Na^+^ have received considerable attention for cathode materials in SIBs. Previous studies on KMFe(CN)_6_ (M = Fe, Mn, Ni, Cu, Co, and Zn) [100] and rhombohedral Na_1.72_MnFe(CN)_6_ [101], have demonstrated that a reversible phase transition incurred upon Na^+^ insertion/extraction. However, these PBAs suffer from poor cyclability and low CE due to their vast vacancies and crystalline water in the lattice, causing large lattice distortions and inefficient Na^+^ storage during cycling. Many strategies have been proposed to optimize crystallization structures, such as high-quality Na_0.61_Fe[Fe(CN)_6_]_0.94_ [102], dehydrated Na_2_MnFe(CN)_6_ [103], single-crystal FeFe(CN)_6_ [104], and high-entropy Na_x_(FeMnNiCuCo)[Fe(CN)_6_] [105], which result in enhanced electrochemical performance of SIB cathodes. Moreover, multiple electron transfer-type Na_2_Mn^II^[Mn^II^(CN)_6_] [106], mesoporous NiFe(II) PBA [107], and cubic Na_x_MnFe(CN)_6_ [108] were reported to achieve high specific capacities, superior rate capability, and excellent cycle stability.

**Fig. 4:**
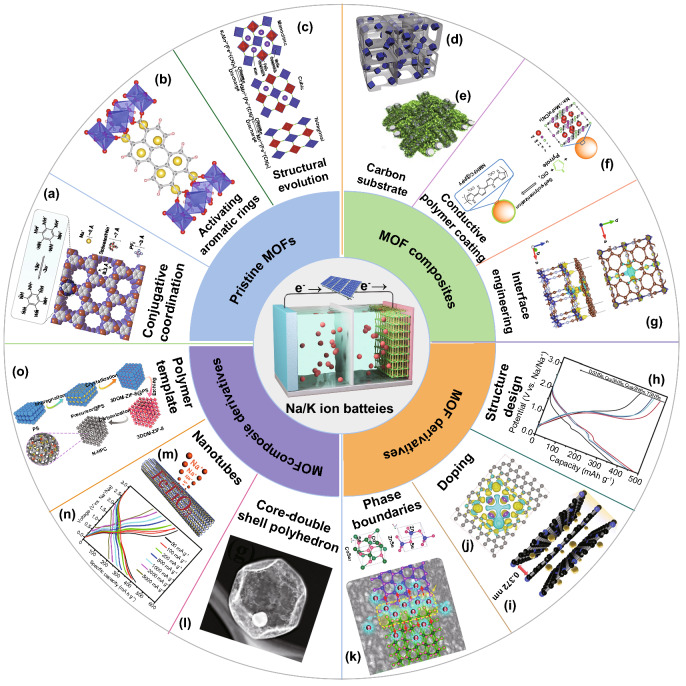
MOF-related materials for SIBs and PIBs. **a** Expected three electron reversible reaction and calculated structure of Co-HAB [110]. Copyright © 2018, American Chemical Society. **b** The most stable position of Na^+^ insertion in Zn-PTCA [111]. Copyright © 2018, Elsevier. **c** Crystal structures and phase transition during charging/discharging processes [145]. Copyright © 2017, Royal Society of Chemistry. **d** Schematic illustration of NaK-MnHCF@3DNC [116]. Copyright © 2019, Elsevier. **e** Schematic illustration of preparation NiCo-MOF cathode [151]. Copyright © 2019, Elsevier. **f** Schematic for synthesis process the NMHFC@PPy preparation [118]. Copyright © 2015, Elsevier. **g** Top and side views of the charge density difference (CDD) of the Co-MOF-RGO composite. The yellow and blue regions refer positive (electron accumulation) and negative (electron depletion) values (in 0.001 e/bohr^3^), respectively [152]. Copyright © 2020, American Chemical Society. **h** Discharge–charge curves of MSNBs with different shell numbers [130]. Copyright © 2018, Wiley–VCH. **i** Schematic illustration K^+^ insertion in NPC [153]. Copyright © 2018, Royal Society of Chemistry. **j** CDD map of four potassium ions embedded into the O/F dual-doped porous carbon [154]. Copyright © 2019, Wiley–VCH. **k** Schematic for the phase-boundary effect in CoZn-Se [135]. Copyright © 2019, American Chemical Society. **l** TEM image of the preparation of ZnS-Sb_2_S_3_@C core-double shell polyhedron [142]. Copyright © 2017, American Chemical Society. **m** Schematic illustration and **n** discharge–charge voltage profiles of FTO ⊂ CNTs [143]. Copyright © 2017, American Chemical Society. **o** Schematic illustration of N-HPC preparation [159]. Copyright © 2019, American Chemical Society

MOFs were also reported as anode materials for SIBs recently. It was demonstrated that the ultrathin cobalt terephthalate-based MOF nanosheets (u-CoOHtp) with oxygen vacancies could induce a local built-in electric field, which is beneficial for accelerating ion diffusion rate and thus improve the reversible Na^+^ storage [109]. To enhance the conductivity of the electrode material, a novel cobalt-based 2D conductive MOF (Co-HAB, consisted of Co(II) ion node and redox active hexaaminobenzene (HAB) linker) has been proposed for SIBs (Fig. 4a) [110]. As a SIB anode material, Co-HAB presents a high rate capability of 214 mAh g^−1^ within 7 min or 152 mAh g^−1^ in 45 s, corresponding to a redox process of three electrons. Besides, Huang and co-workers designed a stable 3D wavy-layered structure of MOF, zinc perylenetetracarboxylates (Zn-PTCA), which enables aromatic rings activated as sodium storage sites (Fig. 4b) [111]. Such Zn-PTCA anode achieves a high discharge capacity of 357 mAh g^−1^ at a rate of 50 mA g^−1^, corresponding to the eight electrons transfer process. This work can provide an efficient strategy to design 3D MOF structures for high-capacity electrode materials.

### MOF Composites

In many cases, as a MOF material, low conductivity and poor structural stability seriously limit the electrochemical performance of PB and PBAs. To tackle these issues, several unique nanostructures of MOF composites with conductive carbon, such as Ketjen black [112], CNTs [113], and graphene [114], have been developed. In particular, the 3D conductive carbon networks have attracted special attention on account of their high specific surface area, excellent electrical conductivity, and superior thermal/chemical stability. For example, a metal–organic cuprous tetracyanoquinodimethane in situ grown on 3D carbon nanofiber network (CuTCNQ/CNFs) has been prepared and used as a freestanding cathode for SIBs [115]. The highly interconnected CNFs enabled rapid electron transfer and maintained the integrity of the electrode during cycling. In another case, a 3D cathode material for SIBs was fabricated by compounding Na_*x*_K_*y*_MnFe(CN)_6_ (*x* + *y* ≤ 2, NaK-MnHCF) with hierarchical porous 3D N-doped ultrathin carbon networks (3DNC) (Fig. 4d) [116]. The 3DNC not only provides large specific surface and abundant active sites to inhibit the aggregation of NaK-MnHCF particles, but also enhances the electrical conductivity of the NaK-MnHCF, which boosted the sodium storage of the NaK-MnHCF@3DNC composite.

Another possible strategy is to integrate MOFs with conductive polymers, especially the polypyrrole (PPy) [117]. Dou and co-workers synthesized ClO^4−^ doped conducting polypyrrole-coated Na_1+*x*_MnFe(CN)_6_ composite (NMHFC@PPy) for SIBs cathode material via a simple and one-step chemistry approach (Fig. 4f) [118]. The authors only used the intrinsic oxidation capacity of the NMHFC to form conductive PPy on the surfaces of NMHFC particles. In this study, PPy plays multiple significant roles in the overall electrochemical performance of the NMHFC@PPy composite. Specifically, PPy as a conducting polymer can improve the electronic conductivity of NMHFC to enhance the rate capability. Next, PPy can act as a protective layer to prevent the dissolution of Mn in the NMHFC structure to increase the cycling stability. Finally, the PPy doped with ClO^4−^can provide redox active sites to improve the capacity of the NMHFC@PPy composite.

### MOF Derivatives

MOF-derived carbon materials with porous structure, abundant heteroatoms doping, and desirable electrical conductivity have exhibited remarkable application prospects for SIBs. In this regard, Qu et al. prepared ZIF-8-derived microporous carbon (ZIF-C) with a uniform pore size of 0.5 nm. ZIF-C exhibited a higher capacity and superior reversibility than mesoporous carbon (CMK-3) [119]. Also, Zhang’s group developed sulfur-doped mesoporous carbons via pyrolysis and sulfuration of MOF-5 [120]. Sulfur doping can enlarge the interlayer distance of carbon and provide more active sites for sodium storage. It is important to develop MOF-derived carbon anode with desirable morphology, especially the 2D ones. Therefore, Liu et al. synthesized 2D Zn-hexamine MOF-derived N-rich porous carbon nanosheets (NPCNs) as an anode material for SIBs [121]. The as-prepared 2D NPCNs can provide large surface area and abundant accessible active sites for superior sodium storage. As a result, a high reversible capacity (318 mAh g^−1^ at 100 mA g^−1^) and ultrafast sodium storage capability (194 mAh g^−1^ at 10 A g^−1^) were achieved by these carbon nanosheets. In addition, carbon/metal hybrid resulting from transformation of single MOF can improve the sodiophilic for sodium nucleation/deposition and accommodate huge volume change, achieving the dendrite-free Na metal anodes [122, 123].

MOF-derived metal oxides such as TiO_2_ [124], Co_3_O_4_ [125], and V_2_O_3_ [126] are considered to be attractive anode materials for SIBs because of their high specific capacities and environmental friendliness. However, they may suffer from poor rate performance and cycling stability owing to low electrical conductivity and poor structure stability during the charging/discharging process. Transforming MOFs into metal sulfides, phosphides, and selenides with high electrical conductivity and desirable architecture/components would be favorable [127–129]. Lou’s group developed ZIF-67-derived CoS_2_ multi-shelled nanoboxes through solvothermal treatment with complex anion conversion and exchange reaction [130]. The CoS_2_ with double, triple, quadruple, and quintuple shells (denoted as CoS_2_ with DSNBs, TSNBs, QuaSNBs, and QuiSNBs, respectively) delivered high discharge capacities of 478.1, 449.6, 442.2, and 380.0 mAh g^−1^ at 500 mA g^−1^, respectively (Fig. 4h). The as-prepared triple-shelled CoS_2_ nanoboxes maintained a high specific capacity of 438 mAh g^−1^ over 100 cycles at 500 mA g^−1^. The multi-shelled nanostructures not only provided large specific surface area and adequate active sites for sodium storage, but also alleviated the volume expansion during sodiation/desodiation process, which enhanced the overall electrochemical performance of the CoS_2_ multi-shelled nanoboxes. In addition to the conversion-type anodes, MOF-derived alloy-type anodes have been developed for fast and efficient sodium storage [131]. Recently, Bi-MOF is loaded with Sb salts within its pores and is applied as the precursor for the preparation of Bi–Sb alloy nanoparticles by the nanoscale laser metallurgy strategy [132]. The obtained Bi_0.70_Sb_0.30_ anode exhibited excellent rate performance and cycle stability due to 1D nanostructure with proper Bi/Sb atomic ratios, effectively facilitating the permeation of electrolyte and movement of electron/ion, as well as the mitigation of volume expansion.

Compared to single MOF derivatives, dual MOF derivatives possess higher electrical conductivity and richer redox reactions due to their abundant phase boundaries. Previous works have demonstrated that MOF-derived bimetal compounds could accelerate reaction kinetics, thus enhancing sodium storage performance [133, 134]. As an example, a bimetallic selenide heterostructure (CoSe_2_/ZnSe) was developed through a facile oil-bath treatment and selenization of 2D CoZn-MOFs [135]. It is documented that heterointerface with high electron density in ZnSe crystalline side is more favorable to the adsorption of Na^+^ ions (Fig. 4k), therefore accelerating reaction kinetics for SIBs. The excellent capacity retention of 93% after 100 cycles was achieved for CoZn-Se anode, while the mono-metal selenides exhibit fast decay in capacity.

### MOF Composite Derivatives

Constructing MOF composite derivatives could provide an opportunity to achieve synchronous advantages between different components. The MOF/carbon (i.e., CNTs, graphene, and carbon cloth) composite derivatives have been widely applied as anode materials for SIBs [131, 136, 137]. Chen et al. prepared N-doped porous carbon nanocomposites (NPCNs) derived from ZIF-8 and carbon (1D CNT and/or 2D graphene) composites [138]. Because of the synergistic effect of N-doped porous carbon, CNTs and graphene, the optimized NPCNs exhibited superior sodium storage performance among the other NPCNs. In another case, Co, N-doped mesoporous TiO_2_/C frameworks transformed from the Ti-MOFs/graphene oxide composite were developed to boost fast sodium storage [124]. The high reversible capacities of 174, 121, and 100 mAh g^−1^ were yielded at 6, 15, and 30 C for over 5000, 10,000, and 3000 cycles, respectively. The dual-doping incorporates abundant oxygen vacancies into the TiO_2_ nanoparticles, significantly enhancing their electrical conductivity. The interwoven graphene porous networks facilitate electron conduction and Na^+^ transport through the overall electrode.

The development of MOFs/polymeric composite derivatives is of great importance in order to fabricate anode materials for SIBs [139]. In one interesting study, a bimetallic Zn-Co-ZIF shell was formed by fixing two Co(Ac)_2_ and Zn(Ac)_2_ into polyacrylonitrile (PAN) electrospun nanofibers [140]. The subsequent annealing in inert gas under 700 °C, the Zn-Co-ZIF shell converted into well-graphitized carbon, while the carbon core (carbonized from the PAN) was etched by ZIF-8-derived ZnO nanoparticles. The as-prepared N-doped carbon hollow tubules exhibited a high reversible capacity of 346 mAh g^−1^ and ultralong cycling life over 10,000 cycles. Similarly, nitrogen- and oxygen-doped porous carbon nanofibers (PCNFs) were synthesized through thermal decomposition of the bimetallic ZnNi-MOF/PAN electrospun nanofibers [141]. The PCNFs can reduce the Na adsorption energy barrier as well as enhance the nucleation and deposition of Na, which can achieve the dendrite-free and ultrastable Na metal anodes. Polymers engineered on the surface of MOFs can also serve as a stabilization layer for the composite derivatives. Yin’s group developed a resorcinol–formaldehyde (RF)-coated ZIF-8 polyhedron composite [142]. After the sulfurization with thioacetamide (TAA) and the cation exchange process with Sb^3+^, a ZnS-Sb_2_S_3_@C core–double-shell polyhedron was finally obtained (Fig. 4l). When evaluated as alloy-type anode for SIBs, the ZnS-Sb_2_S_3_@C exhibited a high specific capacity of 630 mAh g^−1^ after 120 cycles at 100 mA g^−1^.

Metal compound/MOF composite derivative is another effective strategy for constructing multicomponent electrode materials. Ilmenite FeTiO_3_ nanoparticles embedded in carbon nanotubes (FTO ⊂ CNTs) were synthesized by a facile annealing process of TiO_2_ coating Fe-MOF nanorods (Fig. 4m) [143]. Benefiting from the distinct advantages of hollow 1D nanostructure, ultrafine electroactive sites, and flexible conductive carbon substrate, FTO ⊂ CNT electrode achieved high capacities for sodium storage (Fig. 4n). Besides, Kang and co-workers fabricated ZIF-67 shells on the MoO_3_ nanobelts, followed by the sulfidation process [144]. The obtained CoMoS_3_ nanobackbones were coated with polydopamine and then carbonized under inert conditions, leading to the generation of CoMoS_3_@N-doped carbon nanobackbones (CoMoS_3_@NC). The CoMoS_3_@NC anode delivered improved sodium storage performance because of their hierarchical nanostructure, conductive N-doped carbon shell, and the synergistic effect between multiple components.

### Summary

MOF/MOF composite cathodes and MOF/MOF composite-derived anodes have been regarded as promising candidates for electrode materials in SIBs. Although great progress on the development of MOF electrode materials for SIBs has been demonstrated, several challenges still exist and limit their electrochemical applications. The low electrical conductivity, structural instability, and the low tap density of MOFs lead to poor rate capability, low cycling stability, and volumetric energy density. These challenging issues may be addressed to some extent by the aforementioned approaches. However, more efforts need to be done to explore storage mechanisms and optimize the structure, compositions, and properties of MOF materials in SIBs.

## Potassium-Ion Batteries

### Pristine MOFs

Apart from the applications as electrode materials for SIBs, MOFs could be applied for PIBs. Komada and co-workers proposed Prussian blue analogues, K_1.75_Mn[Fe(CN)_6_]_0.93_·0.16H_2_O (K-MnHCFe), and K_1.64_Fe[Fe(CN)_6_]_0.89_·0.15H_2_O (K-FeHCFe), as affordable cathode materials for PIBs (Fig. 4c) [145]. In particular, K-MnHCFe displayed a high capacity of 141 mAh g^−1^ and good cycling stability because of its open and flexible framework structure. Recently, Chen and co-workers proposed a low-strain potassium-rich K_1.84_Ni[Fe(CN)_6_]_0.88_∙0.49H2O (KNiHCF) as a cathode material for PIBs [146]. The KNiHCF exhibited an excellent rate performance (45.8 mAh g^−1^ at 5000 mA g^−1^) due to the low K^+^ diffusion barrier. Besides, a vanadium-based MOF material K_2_[(VO)_2_(HPO_4_)_2_(C_2_O_4_)] with large interplanar lattice spacing was synthesized as a cathode for PIBs [147]. Highly reversible K^+^ extraction/insertion in the layers was realized, with good cycling stability (capacity retention of 83%) after 200 cycles.

MOFs are also promising anode materials for PIBs because of their abundant electroactive components and regular ion diffusion channels. For example, a MOF (MIL-125(Ti)) with high porosity, unique ligand, and low toxicity was synthesized and probed as the anode material for PIBs [148]. Benefiting from porous structure, active carboxylate groups, and reversible potassiation/depotassiation process in MIL-125(Ti), the electrode delivered a high-capacity retention of 90.2% over 2000 cycles with a Coulombic efficiency of 100%. Hu’s group reported cobalt(II) terephthalate-based layered MOF (L-Co_2_(OH)_2_BDC, BDC = 1,4-benzenedicarboxylate) as an anode material for PIBs with high reversible capacity of 246 mAh g^−1^ at 100 mA g^−1^ and excellent cycling stability (188 mAh g^−1^ after 600 at 1 A g^−1^) [149]. They found that coordination between cobalt and oxygen ions greatly ensures a reversible K^+^ insertion/deinsertion process.

### MOF Composites

Among the major issues that limit MOF applications are both its low stability and poor electrical conductivity. The fabrication of nanocomposites between MOFs and various carbonaceous materials is an effective way to improve the stability and electrical conductivity of MOF. The single-walled carbon nanotubes (SWCNTs) or multi-walled carbon nanotubes (MWCNTs) have been demonstrated as a reactant to the electrosynthesis of PB, yielding CNT/PB nanocomposite thin films as cathodes for PIBs [150]. In another case, a Ni/Co-oxygen octahedron layers pillared by NiCo-2,6-NDC nanosheets were grown on carbon cloth as a good candidate for PIBs (Fig. 4e) [151]. In this MOF, the carboxylate layer enlarges the interplanar space for fast ion transportation to expose Ni and Co redox centers, which makes great contributions to its high capacity and excellent rate performance (225 mAh g^−1^ at 1 A g^−1^ and 185 mAh g^−1^ at 20 A g^−1^). Recently, Xu and co-workers constructed Co-MOF nanocrystals encapsulated in a 3D graphene network (Co-MOF-RGO) via strong chemical interaction as freestanding anodes for PIB (Fig. 4g) [152]. It is demonstrated that the strong chemical-bonded interface can significantly enhance charge transfer, adsorption, and diffusion of the potassium ion within the MOF nanocrystals compared to the physical mixture of Co-MOF nanocrystals and reduced graphene oxide (RGO).

### MOF Derivatives

MOF-derived carbon materials and metal compounds/carbon composites have been demonstrated impressive performances in PIBs. Compared with MOFs, MOF-derived porous carbon materials are very attractive for PIBs anodes due to their high conductivity, abundant surface defects, and stable carbon skeleton structure. For this, Li et al. prepared high pyridine N-doped porous carbon (NPC) derived from the ZIF-67 as an anode material for PIBs [153]. The high content of pyridinic N and negligible change of interlayer space (Fig. 4i) can offer additional adsorption sites of K^+^ and thus ensure structure stability. Therefore, the NPC-600 can deliver a high reversible capacity (587.6 mAh g^−1^ at 50 mA g^−1^) and long lifespan (231.6 mAh g^–1^ at 500 mA g^–1^ after 2000 cycles). Multiple heteroatoms’ doping is also a promising strategy to enhance the electrochemical properties of carbon materials. A kind of oxygen/fluorine dual-doped porous carbon nanopolyhedra (OFPCN) was synthesized from carbonization, etching and annealing UiO-66 (Zr) MOF as a novel anode material for PIBs [154]. The obtained OFPCN electrode achieved a high specific capacity of 481 mAh g^−1^ at 0.05 A g^−1^ and ultralong cycling stability of 111 mAh g^−1^ over 5000 cycles at 10 A g^−1^. Oxygen/fluorine co-doping can effectively tune the electronic structure of carbon atoms and enhance the K atoms adsorption ability (Fig. 4j), which could account for such excellent performance of the OFPCN electrode.

MOF-derived metal species/carbon has been developed and used as electrode materials for PIBs because of its desirable conductivity, high theoretical capacity, and good electrochemical activities. The Co_0.85_Se nanoparticles embedded in N-doped carbon polyhedrons (Co_0.85_Se-NC) were prepared by carbonization and selenization of ZIF-67 [155]. Due to the uniform distribution of Co_0.85_Se together with the high specific surface area from mesoporous structures and improved electric conductivity of N-doped carbon, the Co_0.85_Se-NC exhibited a specific capacity of 114.7 mAh g^−1^ after 250 cycles at 1000 mA g^−1^. Besides, Lu’s group reported ultrathin carbon film@carbon nanorods@Bi nanoparticle (UCF@CNs@BiN) composites by pyrolysis of Bi-MOFs as anodes for PIBs [156]. The UCF@CN matrix can not only direct most solid electrolyte interphase (SEI) film formation on the carbon film surface, but also provide a fast channel for ion transport and accommodate the volume variation of Bi nanoparticles during many potassiation/depotassiation cycles. As a result, the UCF@CNs@BiN anodes delivered an outstanding capacity of 425 mAh g^−1^ at 100 mA g^−1^ and a capacity decay rate of 0.038% per cycle after 600 cycles.

### MOF Composite Derivatives

Building elaborately designed composites of MOF with carbon or polymer material and the subsequent conversion to metal species/carbon derivatives are effective approaches in achieving excellent potassium storage performance. For example, Mai and co-workers proposed MOF-74/graphene oxide composite-derived NiCo_2.5_S_4_ microrods wrapped in reduced graphene oxide (NCS@RGO) for potassium-ion storage [157]. The introduction of RGO enhanced excellent electrical conductivity and fast K^+^ diffusion kinetics in the NCS@RGO. Moreover, NCS@RGO electrode with organic potassium salt-containing electrolyte reduced byproduct formation and enhanced the mechanical stability of electrode due to the formation of a robust SEI layer. The NCS@RGO anode displayed a high initial reversible capacity (602 mAh g^−1^ at 50 mA g^−1^), excellent rate capability (402 mA h g^−1^ at 2 A g^−1^), and ultralong cycle life (495 mAh g^−1^ at 200 mA g^−1^ after 1900 cycles).

Electrospinning MOF/PAN nanofiber derivatives have also been developed for PIBs. As an example, Zhang’s group reported a simple method for the preparation of MOF/PAN composite by using a mixture of ZIF-67 nanocubes, PAN, and DMF as electrospun precursors [158]. The subsequent carbonization–selenylation process led to the confinement of Co_0.85_Se@C nanoboxes within carbon nanofibers (Co_0.85_Se@CNFs). In this derivative, Co_0.85_Se@C nanoboxes with high surface area and adequate void space alleviate the volume expansion for improved cycling stability. Furthermore, the robust CNFs network enhanced the electronic conductivity and stabilized the integral structure upon repeated potassiation/depotassiation process. As a result, this unique nanoarchitecture exhibited good cycling stability (353 mAh g^–1^ at 0.2 A g^–1^ after 100 cycles) as an anode material for PIBs.

Polystyrene (PS) spheres are commonly utilized templates for the fabrication of porous or hollow structures by removed PSs. Yu and co-workers fabricated 3D MOF/PS composite for PIBs by in situ growth of ZIF-8 between the clearances of 3D PS monolith (Fig. 4o) [159]. Subsequently, the hierarchical porous carbon with N-doped (N-HPC) was obtained by removed PS template and heat-treated at 900 °C in Ar atmosphere. The as-prepared N-HPC anode achieved high reversible capacity (292 mAh g^−1^ at 0.1 A g^−1^), superior rate performance (94 mAh g^−1^ at 10.0 A g^−1^), and extraordinary long cycle life (157 mA g^−1^ after 12,000 cycles at 2.0 A g^−1^). The 3D hierarchical porous structure reduced the transportation distance for both ions/electrons, while N-doping enhanced the active sites and electronic conductivity through forming more defects. Moreover, the bicontinuous structure with a high specific surface area could decrease the current density and enhance the rate performance.

### Summary

Applying MOFs as cathodes and MOF derivatives as anodes for PIBs has been demonstrated in recent years. However, they may suffer from several issues, such as structural instability, intrinsically high surface area, and poor electric conductivity, which could result in poor cycling stability, low initial CE and volumetric energy density, and inferior rate capability. It is highly desirable to achieve symmetry among the porosity, structure, and conductivity for applications of MOF-based electrodes in PIBs. In addition, the detailed structural transformation and potassium storage processes of MOF-based materials are still unclear. These challenges may be mitigated to some extent by the aforementioned strategies. However, more research in this area needs to be done to reveal and optimize the basic electrochemical mechanism by in situ characterizations and theoretical simulations and further optimize MOF-based materials’ potassium-ion storage performances.

## Aqueous Zn-Ion Batteries

### Pristine MOFs

**Fig. 5:**
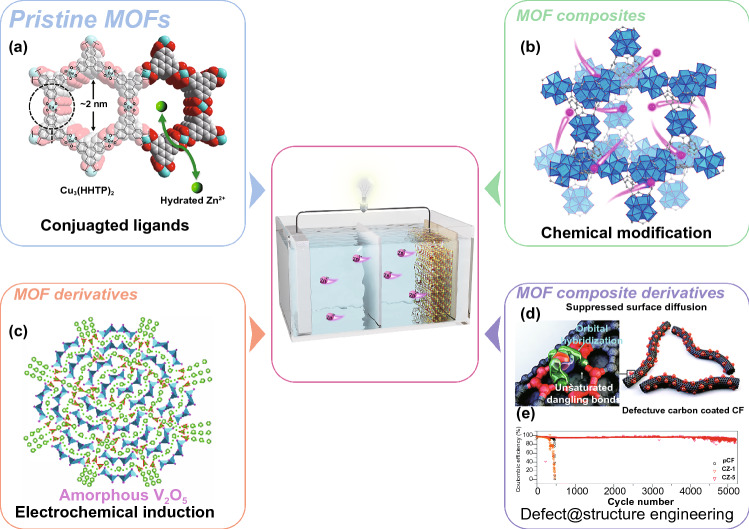
MOF-based materials for ZIBs. **a** Crystal structure (the cyan, red, and gray spheres represent Cu, O, and C atoms, respectively) of Cu_3_(HHTP)_2_ [164]. Copyright © 2019, Nature Publishing Group. **b** Crystal structure of ZnMOF-808. Blue polyhedrons represent Zr-O clusters and pink balls represent Zn^2+^ ions [168]. Copyright © 2019, Elsevier. **c** Schematic illustration of Zn^2+^ diffusion pathways and Zn^2+^ (de)intercalation energy in amorphous V_2_O_5_ [170]. Copyright © 2020, Wiley–VCH. **d** Schematic of uniform Zn deposit and lateral growth on the defective carbon layer-coated 3D CF. **e** Columbic efficiencies of pristine CF (Pcf), non-defective CF (CZ-1), and defective CF (CZ-5) [175]. Copyright © 2020, Royal Society of Chemistry

Among the various cathode materials for ZIBs, PBAs have been investigated due to their open framework structures, which could contribute to the excellent cycling performance [160–162]. As an example, Liu and co-workers found that zinc hexacyanoferrates (Zn_3_[Fe(CN)_6_]_2_, ZnHCFs) can provide reversible insertion/extraction of Zn^2+^ and keep stable in aqueous ZnSO_4_ electrolytes [163]. Consequently, the Zn/ZnSO_4_/ZnHCF battery delivered an average operation voltage of 1.7 V, good rate capability (32.2 mAh g^−1^ at 20 C), and cycle stability (the capacity retention of 81% after 100 cycles). Besides, Stoddart’s group reported a conductive 2D Cu_3_(HHTP)_2_ (HHTP = 2,3,6,7,10,11-hexahydroxytriphenylene) with large open channels as cathode for ZIBs (Fig. 5a) [164]. They found that hydrated Zn^2+^ ions can insert directly into the Cu_3_(HHTP)_2_ pores, allowing fast diffusion rate and small interfacial resistance, which provide enhanced rate capability and cyclability. Moreover, both copper and the quinoid structures in Cu_3_(HHTP)_2_ can serve as redox active sites to increase the specific capacity of the cathode material. The reversible shifts of (100) peaks in XRD and the high capacitive contribution suggest that Cu_3_(HHTP)_2_ follows an intercalation pseudocapacitive charge storage mechanism. As a result, Cu_3_(HHTP)_2_ achieved a high reversible capacity of 228 mAh g^−1^ at 50 mA g^−1^ and capacity retention of 75.0% over 500 cycles at a high current density of 4000 mA g^−1^.

Developing a regular porous MOF coating on Zn anode can also guide homogenous deposition and suppress Zn dendrite formation. For example, MOF (ZIF 7) was coated on Zn anode surface as a super-saturated electrolyte layer for stable aqueous ZIBs [165]. ZIF 7 channels (2.94 Å) would reject large-sized charged ion complexes and form a super-saturated electrolyte in ZIF 7 channel under electric field. Moreover, MOF-coated Zn anode exhibited round-edged and dense-packed electrodeposits without byproduct accumulation. Taking advantage from the super-saturated front surface, symmetric Zn cells delivered ultralong cycle life over 3000 h at 0.5 mA cm^−2^. When fabricated with MnO_2_ cathode, ZIBs achieved a high reversible capacity (180.3 mAh g^−1^) and excellent cycle stability (capacity retention of 88.9% after 600 cycles) under MnO_2_ loading of 4.2 mg cm^−2^. Recently, Pu et al. reported all-MOF-based integrated high-performance aqueous ZIBs constructed by Mn(BTC) MOF cathodes and ZIF‑8‑coated Zn (ZIF‑8@Zn) anodes [166]. The unique porous structure of ZIF‑8 coating was applied to protect Zn metal anodes, which resulted in a uniform electrolyte flux and effectively suppressed the formation of Zn protuberances/dendrites.

### MOF Composites

MOF composites incorporating highly conductive carbon materials can improve MOF-based cathode performance in ZIBs. 3D conductive vanadium-based MOFs (V-MOFs, MIL-47) nanowire-bundle arrays were recently developed by growing on carbon nanotube fibers (CNTFs) as binder-free cathodes for ZIBs [167]. During the synthetic process, V-MOF nanowire-bundle arrays were engineered on the surface of CNTFs, which were then immersed into the VOSO_4_ and p-phthalic acid-containing DMF solutions for the hydrothermal reaction. By virtue of their desirable conductivity, rich active sites, and hierarchical porosity, a fiber-shaped V-MOF-CNTFs cathode displayed an excellent volumetric capacity of 101.8 mAh cm^−3^ at 0.1 A cm^−3^ and superior rate capability (65.5 mAh cm^−3^ at 5.0 A cm^−3^).

Modification strategy can construct MOF functional composites to provide fast ion transportation and protect Zn metal anode. Pan’s group described a single-ion Zn^2+^ solid-state electrolyte (SSE) through a post-synthetic modified MOF-808 ([Zr_6_O_4_(OH)_4_(HCOO)_6_(BTC)_2_]) [168]. Specifically, MOF-808 was turned negative by HCl treatment to obtain HMOF-808 (Fig. 5b). The counter H^+^ ions in the pores were replaced by Zn(H_2_O)_6_^2+^ by immersing HMOF-808 in zinc acetate solution. Owing to the confined highly concentrated Zn(H_2_O)_6_^2+^ ions within pores of MOF host, water@ZnMOF-808 (WZM) SSE demonstrates high ionic conductivity (2.1 × 10^−4^ S cm^−1^ at 30 °C), low activation energy (0.12 eV), and high Zn^2+^ transference number (0.93). WZM-SSE also shows good compatibility with Zn metal anode and stable Zn plating/stripping free from dendrite by virtue of its solid microporous structure with nanowetted SSE/Zn metal interface. The VS_2_/SSE/Zn batteries exhibited a reversible capacity of 125 mAh g^−1^ after 250 cycles at 0.2 A g^−1^.

### MOF Derivatives

Metal compounds are often mixed with carbon materials to assemble the conventional cathodes of ZIBs because of their poor conductivity. However, the connections between metal compounds and conductive carbon are usually just the simple physical contact in traditional cathodes, limiting the rapid electron transport and thus resulting in poor rate performance. MOF derivatives with uniform distribution of metal compounds and carbon would hold great promise to achieve high-performance ZIBs [169]. As an example, Niu and co-workers developed a MIL-88B(V)-derived amorphous V_2_O_5_/carbon material (a-V_2_O_5_@C) by carbonization and in situ electrochemical induction strategy (Fig. 5c) [170]. The amorphous V_2_O_5_ enables more isotropic Zn^2+^ diffusion routes and reaction sites, leading to a faster Zn^2+^ diffusion than that of crystalline V_2_O_5_ (c-V_2_O_5_). Moreover, the porous carbon framework offers a continuous electron transfer pathway and ion diffusion channels. As a result, the a-V_2_O_5_@C ZIB cathode exhibits superior rate performance compared with a-V_2_O_5_ and c-V_2_O_5_ materials.

MOF derivative-based Zn anodes have been presented recently. Wang and co-workers reported ZIF-8-derived Zn/carbon nanoparticles as a host matrix for electrodeposited Zn [171]. The optimized MOF derivative (ZIF-8 annealed at 500 °C, ZIF-8–500) is an attractive host material for highly stable CE (close to 100%) and dendrite-free Zn plating and stripping behavior. Moreover, I_2_//Zn@ZIF-8–500 battery exhibited long cycle stability and good rate capability, which is a great improvement compared to the I_2_//Zn cell. The excellent electrochemical performances can be attributed to abundant porous structure and amount of Zn species in the ZIF-8-500 framework, which offers homogeneous nuclei for Zn plating and large overpotential for hydrogen evolution to alleviate H_2_O decomposition side reaction. MOFs can also be directly grown on Zn metal, whose derivatives can be utilized as integrated anodes for ZIBs. Ruoff’s group engineered ZIF-8 layer directly on the surface of Zn foil by successively dipping foil in an ammonium persulfate solution and 2-methylimidazole solution, and the derived Zn/carbon layer on Zn foil was demonstrated to be an excellent integrated anode for ZIBs [172]. The MOF-based integrated anode suppressed Zn dendrite formation and side reactions due to its hydrophilic and porous surface. Thus, ZIBs with modified Zn anode achieved improved performance including reversible capacity, rate capability, and cycle stability relative to the pure Zn anode.

### MOF Composite Derivatives

Combining cathode material with MOF to form composite derivative could be an effective strategy for improving the overall performance of ZIBs. In one example, ZIF-8 nanoparticles were fabricated on the surface of polyvinylpyrrolidone-modified MnO_2_ nanorods by Sun and co-workers to obtain a robust composite [173]. The subsequent annealing process transformed ZIF-8/MnO_2_ composite into MnO_x_@N-carbon nanorods. With the advantages of abundant porous structure, and conductive carbon shell, the MnO_x_@N–C delivered a much higher capacity of 385 mAh g^−1^ after 120 cycles relative to those of MnO_2_ and M_n_O_y_ cathode materials. In addition, MOF-based materials can be grown on carbon substrates for high-performance ZIBs cathode. For example, flexible carbon cloth was found to be an excellent support for the growth of Mn-MOF nanorod arrays with the solvothermal method. The fabricated Mn-MOF nanorod arrays/carbon cloth composite was then calcinated in the air atmosphere under target temperature, finally obtaining bulk oxygen defects Mn_3_O_4_@C nanorod arrays (O_d_-Mn_3_O_4_@C NA/CC) cathode material, which exhibited better ZIBs performance than the Mn_3_O_4_ nanosheets/carbon cloth electrode [174]. Bulk oxygen defects can change the (MnO_6_) octahedron structure, which enhances structural stability and prevents the dissolution of Mn^2+^. Moreover, the O_d_-Mn_3_O_4_@C NA/CC electrodes also assembled flexible quasi-solid-state ZIBs with high energy density and power density because of the adhesive-free nature.

Constructing MOF/carbon substrate composite derivatives can also serve as a scaffold for dendrite-free Zn deposition. For example, Kim and co-workers proposed a highly porous and defective carbon structure by the direct carbonization of ZIF-8-coated 3D carbon felt (CF) [175]. A single vacancy carbon defect (SV_1_) resulting from the transformation of ZIF-8 prevents the surface diffusion and subsequent aggregation of Zn by inducing a strong orbital hybridization between Zn adatoms and unsaturated dangling bonds of the defect (Fig. 5d). The Zn adsorption energies between the SV_1_ and Zn crystal planes indicated Zn tendency to nucleate on the defect. The consequent growth of Zn nuclei results in the connection of nearby nuclei, coating the whole carbon fiber surfaces and leading to a dense packing of Zn electrodeposits in the 3D carbon framework. As a result, the Zn–Br battery with CZ-5 achieved stable CEs (> 97%) over 5000 cycles (Fig. 5e).

### Summary

Although PBAs as cathode for ZIBs delivered excellent rate and cycling performances, the quite low specific capacity limits their further application. Making more vacancy sites and nanocomposites could be feasible approaches to improve the overall performance of PBAs cathode. Designing electrically conducting MOFs using graphene like extended *π*-conjugation systems is highly desirable for enhanced ZIBs cathode performance. Utilizing MOFs’ modified electrolytes and anodes is also effective strategy for suppressing Zn dendritic formation and enhancing overall performance ZIBs. However, MOFs are chemically unstable for electrochemical applications, especially in acidic/alkaline aqueous conditions. Until now, the development of MOFs for ZIBs remains in its infancy. Thus, efforts must be made to explore MOF-based materials and devise solutions to realize the full potential of MOFs for ZIBs.

## Zn–air Batteries

### Pristine MOFs

**Fig. 6:**
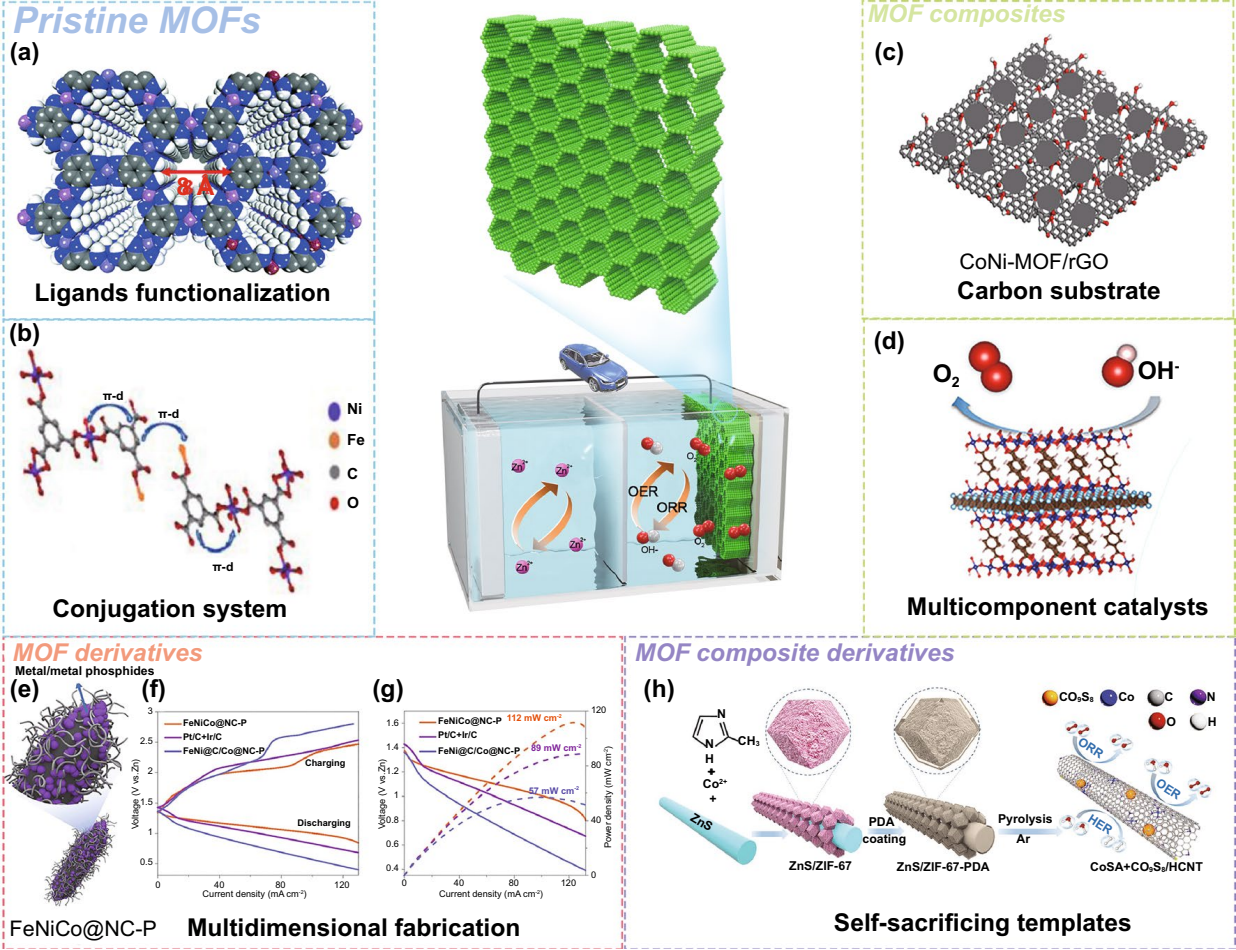
MOF-based materials for Zn–air batteries. **a** Structure illustration of 3D M-HIB-MOFs [176]. Copyright © 2019, Royal Society of Chemistry. **b** Illustration of conjugation system of Ni/Fe-BTC MOFs [177]. Copyright © 2019, Elsevier. **c** Structure illustration of the CoNi-MOF/RGO catalyst [179]. Copyright © 2019, American Chemical Society. **d** Structure of Ti_3_C_2_T_x_ − CoBDC hybrid [182]. Copyright © 2017, American Chemical Society. **e** Schematics of preparation of FeNiCo@NC-P. **f** Charge–discharge profiles, **g** discharge curves and the corresponding power density curves of FeNiCo@NC-P, FeNi@C/Co@NC-P and the mixture of Pt/C + Ir/C, respectively. [189]. Copyright © 2019, Wiley–VCH. **h** Illustration of synthetic strategy of CoSA + Co_9_S_8_/HCNT [27]. Copyright © 2020, Wiley–VCH

As tunable pore structure and abundant active redox sites, MOF is a promising cathode material in the area of rechargeable Zn–air batteries. For example, Lee and co-workers synthesized 3D dual-linked hexaiminobenzene MOF (Mn/Fe-HIB-MOF) hollow spheres by isolated reactions between Mn(II) and Fe(II) nitrates and hexaaminobenzene ligands under room atmosphere, followed by a thermal process (Fig. 6a) [176]. The Mn/Fe-HIB-MOF possesses a high surface area, rapid electron and mass transport pathways, and abundant Mn/Fe–N_4_ active redox sites as compared to conventional MOFs. The Zn–air batteries with Mn/Fe-HIB-MOF exhibited a long cycle life over 6000 cycles (1000 h) at 10 mA cm^−2^ with a narrow voltage gap of 0.75 V.

In another study, Ni/Fe-BTC MOF nanorods were electrochemically prepared via utilizing a two-electrode system containing Ni(II) and Fe(II) chlorides, and BTC linker [177]. When the Ni/Fe-BTC MOF was applied as a cathode in Zn–air batteries, the excellent cycle stability for 5262 cycles (over 594 h) with round trip efficiency (65.80%) at a current density of 10 mA cm^−2^ was delivered. The improved electrochemical performance of Ni/Fe-BTC MOF is attributed to its unique pore structure, intrinsic electrical conductivity, and adequate covalent coordination redox active sites (Fig. 6b).

### MOF Composites

Combining MOFs and conductive carbon is expected to achieve further improved electronic conductivity and catalytic performance in Zn–air batteries. For example, the Co-MOF (Co(bpdc)(H_2_O)_4_ (bpdc = biphenyl–4, 4’-dicarboxylic acid), arrays were in situ grown on a 3D graphite foam (GF) via hydrothermal reaction [178]. The obtained Co-MOF/GF presented excellent bifunctional oxygen activity with a greatly reduced overpotential, which endows the as-assembled Zn–air batteries with good power density and cycling performance. Similarly, a composite with bimetallic CoNi-MOF nanosheets embedded in RGO was also synthesized by a simple mixture of CoNi-MOF and RGO, displaying the adequate exposed active sites and the improved electron conductivity (Fig. 6c) [179]. The Zn–air batteries with CoNi-MOF/RGO-based air electrodes achieved a power density of 97 mW cm^−2^, an energy density of 711 Wh kg_Zn_^−1^, and excellent cycling stability with negligible decline over 120 h. In addition, the direct growth of CoZn-ZIF nanosheet arrays on Ni foam support and its hierarchical porosity can ensure fast electron transfer and mass transportation in Zn–air batteries [180, 181].

2D MXene with high electrical conductivity, surface electronegativity, and good stability are potential substrates that may alter the electrophilicity of the active centers of MOFs and therefore tune their catalytic properties. For instance, Huang and co-workers in situ hybridized cobalt 1,4-benzenedicarboxylate (CoBDC) nanosheets with 2D MXene (Ti_3_C_2_T_x_) by an interdiffusion reaction strategy (Fig. 6d) [182]. The CoBDC layer offered a highly porous structure and large active surface area. The electrically conductive and hydrophilic Ti_3_C_2_T_*x*_ nanosheets enabled the fast charge and ion transfer and promoted the easy access of aqueous electrolytes to the catalytically active CoBDC surfaces. The hybrid Ti_3_C_2_T_*x*_ − CoBDC nanosheets were assembled into air cathodes for Zn–air batteries, exhibiting small overpotential and good cycling stability. Considering the multiple benefits of using MOFs as supports, Cao and co-workers reported grafted Co porphyrins on MOF surfaces through ligand exchange. Zn–air batteries assembled with MOF-supported Co porphyrins catalyst achieved comparable performance to that with Pt/C [183].

### MOF Derivatives

MOF-derived metal-free carbon electrocatalysts with atomically dispersed heteroatoms, abundant porous structure, and high conductivity have been developed as air–cathode for Zn–air batteries. As one example, Zhao and co-workers developed a Zn-MOF, which was synthesized by using 1,4-benzenebicarboxlic acid and sodium tetrakis(1-imidazolyl) borate as organic linkers [184]. During the process of pyrolysis, Zn was evaporated and B, N heteroatoms were retained leading to B-N dual-doped highly porous carbon (BNPC). Benefiting from high porous structure and good oxygen catalytic activities of the N dopants (particularly pyridinic N) and B dopants, the Zn–air batteries with BNPC exhibited initial charge and discharge potentials at 2.19 and 1.16 V (2 mA cm^–2^), respectively, and stable performance even after 100 h (600 cycles).

MOF derivatives with uniformly dispersed metal species and rich nitrogen/carbon sources are also taken into account as significant platform materials in the field of Zn–air batteries [185, 186]. As an example, Zhang et al. synthesized N-doped graphitic carbon with embedded Co nanoparticles (C-MOF-C2-900) transformed from a pair of enantiotopic chiral 3D Co-based MOF ([Co_6_(MIDPPA)_3_(1,2,4-btc)_3_(NO_2_)_3_(H_2_O)_3_](H_2_O)_7_), 1L and 1R, where MIDPPA = 4,4′-di(4-pyridine)-4″-imidazoletriphenylamine and 1,2,4-btc = 1,2,4-benzenetricarboxylic acid, via thermal treatment at temperature 900 °C [187]. The C-MOF-C2-900 with a hierarchical rod-like structure and multiple active sites (metallic Co, CoN_*x*_, CoO_*x*_, and N species) exhibits high electrocatalytic activities for both ORR and OER. The Zn–air batteries based on C-MOF-C2-900 air–cathode delivered excellent initial potentials (1.81 V for charge and 1.28 V for discharge) and stability with almost no decline over 120 h (300 cycles) at 2 mA cm^–2^.

Except for single MOF derivatives, bimetallic MOF and multiple MOF-derived metal species/carbon materials have drawn special attention due to their optimized electronic configuration and enhanced intrinsic activity. Lou and co-workers reported MnO/Co hybrid with porous graphitic carbon (MnO/Co/PGC) polyhedrons by a hydrothermal–calcination strategy with a bimetal MOF containing cobalt and manganese (Co/Mn-MIL-100) as the precursor [188]. Interface engineering of highly active MnO for ORR and high conductive Co for OER endows the MnO/Co/PGC with excellent bifunctional electrocatalytic performance. The Zn–air batteries using the MnO/Co/PGC cathode achieved a high-power density (172 mW cm^−2^) and specific capacity (872 mAh g_Zn_^−1^ at 5 mA cm^−2^) with long-term durability (350 cycles). Moreover, dual-MOFs (Fe_2_Ni_MIL-88@ZnCo_ZIF) were recently developed through self-assembly with PVP-functionalized Fe_2_Ni_MIL-88 (Fig. 6e), Zn(NO)_2_·6H_2_O, Co(NO)_2_·6H_2_O, and 2-methylimidazole in an ethanol solution, the derived CNT-grafted, and N-doped carbon nanorod embedded with Fe–Ni-Co metal/metal phosphide nanoparticles (FeNiCo@NC-P) enhanced the porous structure and intrinsic activity of the oxygen catalysts [189]. The Zn–air batteries with FeNiCo@NC-P realized a low voltage gap and a maximum power density of 112 mW cm^−2^ (Fig. 6f, g).

### MOF Composite Derivatives

During the pyrolysis process, the severe aggregation and structural collapse of MOF derivatives are inevitable, which greatly limit the catalytic performance. Rational construction of MOFs and carbon composite derivatives seem to be an effective strategy in achieving robust bifunctional oxygen electrocatalysts. For example, ZIF-67 was grown on a graphene oxide (GO) substrate, where the composite was then reduced in an H_2_-Ar atmosphere to produce 2D N-doped carbon nanotubes/graphene (GNCNTs) hybrid as an electrocatalyst for Zn–air batteries [190]. The Zn–air batteries assembled by this hybrid catalyst delivered a high power density of 253 mW cm^−2^ and a specific capacity of 801 mAh g_Zn_^−1^, as well as a long lifespan of 9000 cycles (over 3000 h at 5 mA cm^−2^). The superior performance of the hybrid could be ascribed by the hierarchical structure with high specific surface area and rich heteroatoms active sites, which endow high catalytic activity and stability for the oxygen reactions. In addition, Guan et al. proposed a simple solution method to grow Co-MOF arrays directly on the surface of carbon cloth (CC) support [191]. The Co-MOF/CC composite was then converted into hollow Co_3_O_4_ nanosphere-embedded nitrogen-doped carbon arrays (NC-Co_3_O_4_/CC) under pre-carbonized and post-oxidized processes, which exhibited excellent catalytic activities toward both OER and ORR. A solid-state Zn–air battery was assembled using the NC-Co_3_O_4_/CC as a flexible air cathode, which demonstrated high open-circuit potential (1.44 V), satisfactory specific capacity (387.2 mAh g^−1^), and good cycling stability.

The polymer can not only serve as a template to guide structure design, but also be converted into conductive carbon materials to protect unstable electrode materials. Recently, a bifunctional catalyst consisting of Co nanoparticles encapsulated in hollow nitrogen-doped carbon tubes (Co@hNCTs) is synthesized by simple polypyrrole (PPy) tube-directed templating method [192]. Typically, surfactant-treated PPy nanotubes act as the structure-guiding templates for efficient capture of Co^2+^, achieving the in situ growth of ZIF-67 nanocrystals on the surface of PPy nanotubes. The fabricated ZIF-67/PPy nanotubes composite was then carbonized in Ar atmosphere at 800 ℃, finally obtaining Co@hNCTs, which achieved long-term cyclability (over 500 h) when using as an air cathode in Zn–air batteries. Polyacrylonitrile (PAN) is a well-known soft electrospun substrate for designing 1D interconnected N-containing nanofibers. PAN nanofiber film was spun by Guo and co-workers, which was then immersed into Co(NO_3_)_2_ and 2-methylimidazole containing aqueous solution to finally form Co-ZIF on the surface of nanofiber [193]. The subsequent carbonization–acidification process resulted in the formation of Co single-atom supported by N-doped carbon flake arrays grown on carbon nanofibers (Co SA@NCF/CNF). The wearable solid-state Zn–air batteries based on Co SA@NCF/CNF air cathode delivered high specific capacity (530.17 mAh g_zn_^−1^) and superior stability (900 cycles). The excellent performance of the Co SA@NCF/CNF air–cathode stems from the following merits: (1) single-atomic Co sites exhibit low reaction barriers for oxygen catalysis, (2) the hierarchical porous architecture of hybrid endows it to possess adequate accessibility for single atom catalytic sites, (3) the robust carbon nanofiber framework enables high conductivities for rapid electron transfer and provides excellent mechanical properties for structural stability and flexibility in the device.

Developing fabrication strategies of metal compounds/MOF composite derivatives is also important for enhanced oxygen catalysis performance [194, 195]. For example, MnO_2_@ZIF-67 composite was achieved via engineering ZIF-67 shell on the surface of hollow MnO_2_ nanowires [196]. Highly porous MnO@Co–N/C nanowires were then successfully formed after the subsequent pyrolysis process, which exhibited good cycling stability as air–cathode for Zn–air batteries. The excellent performances originate from the synergistic effect of MnO and porous Co–N/C in 1D hollow nanowires, maximizing the catalytic ability of MnO and preventing the aggregation of carbon frameworks. Some metal compounds (i.e., ZnO and SiO_2_) can also be used as self-sacrificing templates for the MOF-based derivatives [185]. Recently, ZnS nanorods were utilized as templates for the growth of ZIF-67 and followed by coating polydopamine (PDA) to obtain ZnS/ZIF-67-PDA composite (Fig. 6h) [27]. Then after pyrolysis at 1000 °C, the ZnS nanorods were reduced to Zn vapors and simultaneously saved as a sulfur source to form Co_9_S_8_ nanoparticles from ZIF-67, finally reaching hollow carbon nanotube embedded single atomic cobalt with Co_9_S_8_ nanoparticles (CoSA + Co_9_S_8_/HCNT). The synergistic effects between Co_9_S_8_ and single atomic cobalt can optimize the electronic structure of the active sites to low the catalytic barrier and promoting the ORR and OER simultaneously. Consequently, Zn–air batteries based on CoSA + Co_9_S_8_/HCNT displayed good stability and a high-power density of 177.33 mW cm^−2^.

### Summary

MOF, MOF composite, and their derivatives have been explored for Zn–air batteries because of their abundant porous structures, operative active sites, and high surface area. However, most of the MOFs suffer from low electrical conductivities and poor stabilities in basic/acidic electrolytes. The incorporation of multi-metal active sites in MOFs or the design of MOF composites should be more encouraged in the next stage of research. In addition, although MOF derivatives possess high conductivities and oxygen catalytic activities, these particles would happen self-aggregation and structural collapse during the operation of Zn–air batteries. It is also important to develop MOF composite derivatives so as to fabricate efficient catalytic materials for high-performance Zn–air batteries. Moreover, MOF-based anodes with hydrophilic and porous surface hold great promise for the development of stable and dendrite-free Zn–air batteries.

## Conclusions and Perspectives

This review provides an overview of recent advances of pristine MOFs, MOF composites, MOF derivatives, and MOF composite derivatives for next-generation batteries (including SIBs, PIBs, ZIBs, AIBs, Li–S, Na–S, Li–Se, Li–O_2_, Na–air, and Zn–air batteries), demonstrating that MOFs are promising candidates for these energy storage applications due to their highly porous structures, controllable morphologies/structures, and tunable chemical compositions. A detailed information of MOFs along with their battery performance parameters is summarized in Table 1.

**Table 1:** Summary of the representative MOF-related materials for next-generation batteries

Materials	MOF utilized	Application	Rate capability (DC^a^/CD^b^)	Reversible capacity (DC/CD)	Cycling Stability (DC/CD/CN^c^)	Refs.
Ni-MOF/S	Ni-MOF	Li–S cathode	287/2 C	689/0.1 C	611/0.1 C/100	[29]
Ni_3_(HITP)_2_	Ni_3_(HITP)_2_	Li–S separator	589/5 C	1244/0.2 C	1139/0.2 C/100	[30]
Cu_2_(CuTCPP)	Cu_2_(CuTCPP)	Li–S separator	437/5 C	1200/0.2 C	1020/0.2 C/100	[31]
HKUST-1	HKUST-1	Li–S separator	633/3 C	1196/0.5 C	802/0.5 C/600	[32]
Ce-MOF-2/CNT	Ce-MOF-2	Li–S separator	663/4 C	1022/1 C	839/1 C/800	[39]
CuBTC-NSP	CuBTC	Li–S separator	963/2 C	1316/0.5 C	1128/0.5 C/500	[45]
B/2D MOF-Co	2D MOF-Co	Li–S separator	478/5 C	1138/0.1 C	703/0.5 C/200	[47]
ppy-S-in- PCN-224	PCN-224	Li–S cathode	640/5 C	680/10 C	440/10 C/1000	[22]
LPS-UiO-66/S	Zr-MOFs	Li–S cathode	–	1040/0.1 C	835/0.2 C/100	[44]
Mg-MOF-74 -PVDF	Mg-MOF-74	Li–S electrolyte	861/2 C	1383/0.1 C	981/0.1 C/200	[49]
CoP@HPCN- MWCNT	ZIF-8/ZIF-67	Li–S cathode	528/3 C	887/0.2 C	753/0.2 C/200	[56]
Co_9_S_8_	2D-MOF	Li–S separator	428/2 C	1385/0.1 C	1190/0.1 C/200	[59]
CP@NCNT@ CoS_3_	Co-MOF	Li–S cathode	–	1601/0.13 C	1047/0.13 C/70	[69]
CC@CS@HPP	ZIF-67	Li–S cathode	–	1223/1 C	1005/1 C/200	[70]
Co/N-PCNSs	Co/Zn-ZIF	Li–S cathode	520/5 C	1234/0.2 C	913/0.2 C/100	[80]
Co_2_B@CNT	ZIF-67	Li–S separator	1137/5 C	1430/0.2 C	1283/0.2 C/200	[78]
Mn-MOF-74	Mn-MOF-74	Li–O_2_ cathode	9420/50 mA g^−1^	–	–	[81]
MnCo-MOF-74	MnCo-MOF-74	Li–O_2_ cathode	–	11,150/200 mA g^−1^	1000/200 mA g^−1^/44	[82]
MOF-74@CNTs	MOF-74	Li–O_2_ cathode	–	2500/125 mA g^−1^	500/125 mA g^−1^/60	[84]
CAU-1-NH2- PMMA	CAU-1-NH2	Li–air cathode	–	1480/200 mA g^−1^	450/450 mA g^−1^/66	[86]
Fe-Fe_3_C@ Fe–N-C	MIL-100(Fe)/ ZIF-8	Li–O_2_ cathode	2878/300 mA g^−1^	8749/50 mA g^−1^	-	[94]
Co-SAs/N–C	Zn-hexamine complex	Li–O_2_ cathode	11,098/1000 mA g^−1^	20,105/200 mA g^−1^	1000/400 mA g^−1^/260	[95]
MOF-C/CNT	ZIF-8	Li–O_2_ cathode	500/600 mA g^−1^	10,050/50 mA g^−1^	500/200/75	[96]
3DP-NC-Co	Co-MOF	Li–O_2_ cathode	525/0.8 mA cm^−2^	1124/0.05 mA cm^−2^	–	[98]
Na_1.72_MnFe(CN)_6_	Na_1.72_MnFe(CN)_6_	SIB cathode	45/4800 mA g^−1^	134/120 mA cm^−2^	120/120 mA g^−1^/30	[101]
Na_0.61_Fe [Fe(CN)_6_]_0.94_	Na_0.61_Fe [Fe(CN)_6_]_0.94_	SIB cathode	70/600 mA g^−1^	170/25 mA g^−1^	170/25 mA g^−1^/150	[102]
cubic Na_x_MnFe(CN)_6_	Cubic Na_x_MnFe(CN)_6_	SIB cathode	74/600 mA g^−1^	124/25 mA g^−1^	84/200 mA g^−1^/500	[108]
u-CoOHtp	u-CoOHtp	SIB anode	215/2000 mA g^−1^	418/50 mA g^−1^	371/50 /mA g^−1^/50	[109]
Co-HAB	Co-HAB	SIB anode	152/12000 mA g^−1^	291/50 mA g^−1^	226/500 mA g^−1^/50	[110]
Zn-PTCA	Zn-PTCA	SIB anode	256/1000 mA g^−1^	450/50 mA g^−1^	302/200 mA g^−1^/50	[111]
CuTCNQ/CNFs	CuTCNQ	SIB cathode	89/600 mA g^−1^	161/300 mA g^−1^	137/300 mA g^−1^/300	[115]
NaK-MnHCF- 3DNC	NaK-MnHCF	SIB cathode	110/500 mA g^−1^	190/40 mA g^−1^	137/40 mA g^−1^/100	[116]
NMHFC@PPy	NMHFC	SIB cathode	56/4800 mA g^−1^	113/240 mA g^−1^	76/240 mA g^−1^/200	[118]
NPCNs	Zn-hexamine MOF	SIB anode	194/10000 mA g^−1^	318/100 mA g^−1^	280/100 mA g^−1^/100	[121]
CoS_2_	ZIF-67	SIB anode	346/5000 mA g^−1^	478/200 mA g^−1^	454/200 mA g^−1^/100	[130]
CoSe_2_/ZnSe	2D CoZn-MOFs	SIB anode	263/10000 mA g^−1^	575/100 mA g^−1^	–	[135]
NPCNs	ZIF-8	SIB anode	146/2000 mA g^−1^	295/100 mA g^−1^	257/100 mA g^−1^/100	[138]
ZnS-Sb_2_S_3_@C	ZIF-8	SIB anode	391/800 mA g^−1^	1043/100 mA g^−1^	630/100 mA g^−1^/120	[142]
FTO ⊂ CNTs	Fe-MOF	SIB anode	202/5000 mA g^−1^	465/100 mA g^−1^	376/100 mA g^−1^/200	[143]
K_2_[(VO)_2_ (HPO_4_)_2_(C_2_O_4_)]	K_2_[(VO)_2_ (HPO_4_)_2_(C_2_O_4_)]	PIB cathode	-	65/21.8 mA g^−1^	54/21.8 mA g^−1^/200	[145]
MIL-125(Ti)	MIL-125(Ti)	PIB anode	56/200 mA g^−1^	155/50 mA g^−1^	157/50 mA g^−1^/200	[148]
L-Co_2_(OH)_2_BDC	L-Co_2_(OH)_2_ BDC	PIB anode	131/1000 mA g^−1^	352/50 mA g^−1^	246/100 mA g^−1^/50	[149]
CC-Ni- NiCo-MOF	NiCo-MOF	PIB cathode	185/20000 mA g^−1^	218/2000 mA g^−1^	–	[151]
OFPCN	UiO-66 (Zr)	PIB anode	78/20000 mA g^−1^	405/100 mA g^−1^	286/100 mA g^−1^/200	[154]
Co_0.85_Se-NC	ZIF-67	PIB anode	111/2000 mA g^−1^	320/50 mA g^−1^	115/1000/ mA g^−1^/250	[155]
UCF@CNs@BiN	Bi-MOFs	PIB anode	140/1000 mA g^−1^	665/100 mA g^−1^	425/100 mA g^−1^/50	[156]
NCS@RGO	MOF-74	PIB anode	402/2000 mA g^−1^	585/50 mA g^−1^	495/200 mA g^−1^/1900	[157]
Co_0.85_Se@CNFs	ZIF-67	PIB anode	166/5000 mA g^−1^	364/200 mA g^−1^	353/200 mA g^−1^/100	[158]
N-HPC	ZIF-8	PIB anode	94/10 mA g^−1^	345/0.1 mA g^−1^	157/2000 mA g^−1^/12000	[159]
Cu_3_(HHTP)_2_	Cu_3_(HHTP)_2_	ZIB cathode	125/4000 mA g^−1^	124/4000 mA g^−1^	93/4000 mA g^−1^/500	[164]
ZIF 7	ZIF 7	ZIB anode	–	192/500 mA g^−1^	187/500 mA g^−1^/180	[165]
ZnMOF-808	MOF-808	ZIB electrolyte	–	140/200	125/200 mA g^−1^/250	[168]
ZIF-8–500	ZIF-8	ZIB anode	80/8000 mA g^−1^	183/200 mA g^−1^	–	[171]
a-V_2_O_5_@C	MIL-88B(V)	ZIB cathode	72.8/200000 mA g^−1^	620/300 mA g^−1^	249/40000 mA g^−1^/20000	[170]
MnO_x_@N–C	ZIF-8	ZIB cathode	–	–	305/500 mA g^−1^/600	[173]
O_d_-Mn_3_O_4_@C NA/CC	Mn-MOFs	ZIB cathode	133/5000 mA g^−1^	396/200 mA g^−1^	84/5000 mA g^−1^/12000	[174]
Mn/Fe-HIB	Mn/Fe-HIB	Zn–air cathode	702/50 mA cm^−2^	769/5 mA cm^−2^	-/10 mA cm^−2^/6000	[176]
Ni/Fe-BTC	Ni/Fe-BTC	Zn–air cathode	706/50 mA cm^−2^	775/10 mA cm^−2^	-/10 mA cm^−2^/5262	[177]
Ti_3_C_2_T_x_-CoBDC	CoBDC	Zn–air cathode	–	–	-/0.8 mA cm^−2^/25	[182]
C-MOF-C2-900	Co-based MOF	Zn–air cathode	741/10 mA cm^−2^	768/5 mA cm^−2^	-/2 mA cm^−2^/360	[187]
MnO/Co/PGC	Co/Mn- MIL-100	Zn–air cathode	–	873/5 mA cm^−2^	-/10 mA cm^−2^/350	[188]
GNCNTs	ZIF-67	Zn–air cathode	728/10 mA cm^−2^	801/5 mA cm^−2^	-/5 mA cm^−2^/9000	[190]
NC-Co_3_O_4_/CC	Co-MOF	Zn–air cathode	–	387/25 mA cm^−3^	-/1 mA cm^−2^/60	[191]

^a^DC: discharge capacity (mAh g^−1^); ^b^CD: current density; ^c^CN: cycle number

**Fig. 7:**
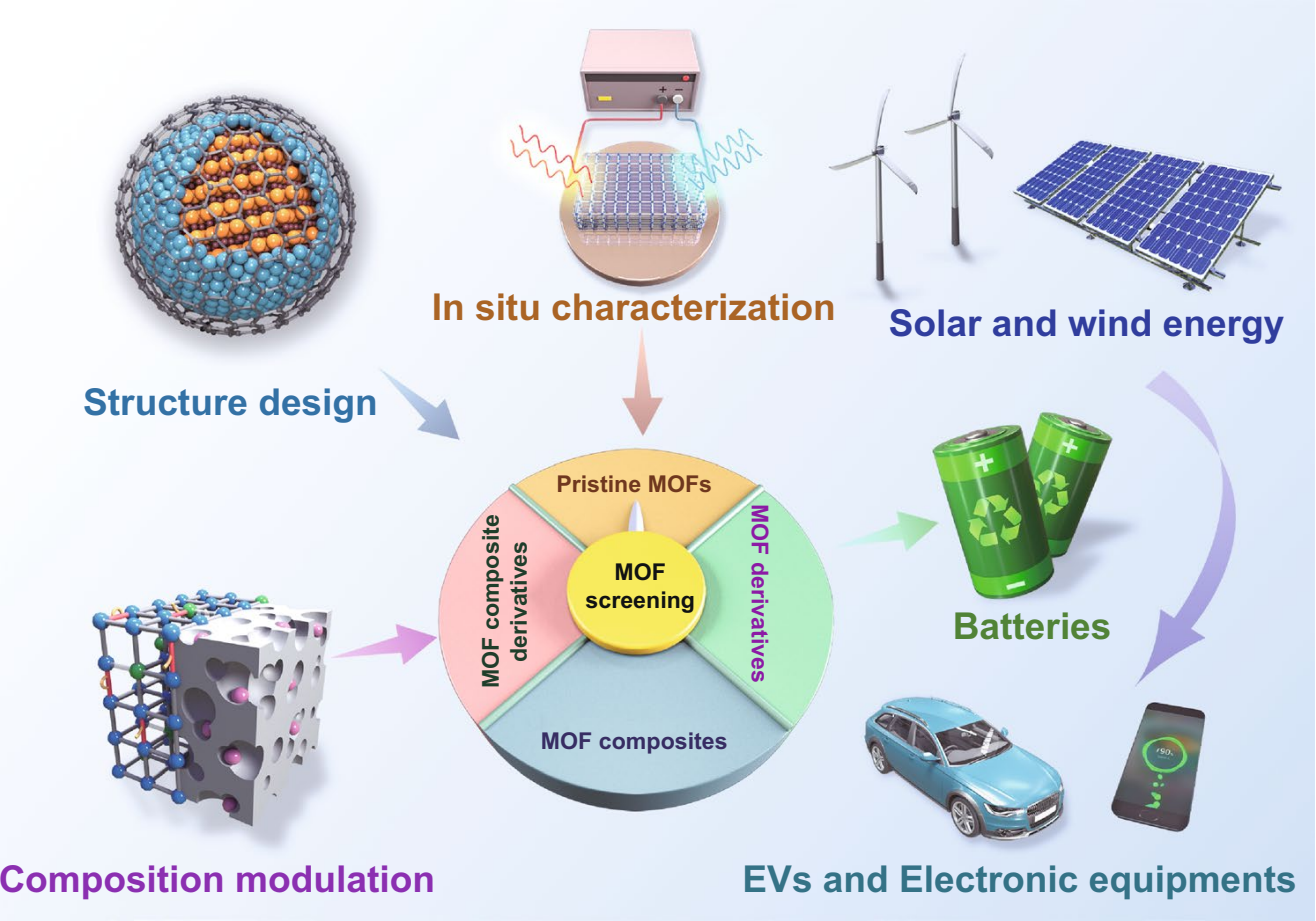
Challenges of MOF-based materials for batteries, mainly including composition modulation and structure design, advanced in situ characterizations, rational MOFs screening, and practical applications

Despite their beneficial features, there are still several issues and challenges on MOFs for various new-generation rechargeable batteries so far (Fig. 7). (1) The poor conductivity and structural stability of pristine MOFs are the major obstacles for battery applications. Although some 2D conductive MOFs have been developed in recent years, they are still unsatisfactory when used as high-rate electrode materials in batteries. Moreover, most of the MOFs suffer from structural collapse, especially in water/moisture, acidic or alkaline environments, which leads to poor cycling stability during the operation of batteries. Apart from developing new MOFs with high conductivity and stability, it is expected that the functionalized strategy by grafting desired atoms/groups or introducing structural stabilizers inside MOFs may also enhance the conductivity or stability of pristine MOFs. Comparatively, MOF derivatives with enhanced conductivity and better stability are considered to be great potential materials for different batteries. However, the self-aggregation and poor microstructures of MOF-derived particles would cause inferior electrochemical performance during the prolonged cycling process in the batteries. In addition, pristine MOFs and their derivatives with high porous structures and large surface areas lead to a low CE and low tap density. Considering these issues, engineering microstructure by combining MOF derivatives with various substrates and adjustment ratio of hybrid electrode components are expected to improve overall battery performances (i.e., CE, gravimetric/volumetric energy density, rate performance, and cycling stability). A significant progress regarding the fabrication of MOF composite and their derivatives has been proposed to achieve better battery performances via the synergistic effect between various components. It should be emphasized that developing efficient functional components, facile and scalable preparation procedures, and low synthetic costs would be the major challenges for MOF composite and their derivatives.

(2) Advanced in situ/operando characterization techniques are anticipated to play more significant roles in exploring the compositional and structural evolution of MOFs and revealing their charge/discharge mechanisms in new-generation batteries. To date, most reported characterizations of MOF-based batteries are ex situ characterizations. Considering the sensitivity of MOFs to air and moisture and variation of complicated battery systems, the realistic electrochemical processes (e.g., dynamic properties, interfacial reactions, and storage mechanisms) may not fully reflect under ex situ measurements. While the in situ characterization techniques could provide real-time information on structural evolution, valence changes, redox reaction mechanism, and SEI formation, during the operation of batteries, for example, in situ X-ray absorption or in situ infrared spectrum is used to investigate the realistic active sites, which would drive an in-depth understanding of MOF-based catalysts in Li–O_2_ batteries and Zn–air batteries. In situ Raman spectroscopy and in situ X-ray absorption can also be employed to investigate catalytic activity of MOFs, revealing the sulfur species conversion kinetics in Li–S batteries.

(3) Rational screening of suitable MOFs (e.g., pristine MOFs/MOF composites and their derivatives) is of great importance for targeted next-generation battery applications. For Li–S batteries, MOFs consisting of Lewis acidic metal center ions and Lewis basic organic ligands are desirable for sulfur storage and polysulfides immobilization. MOF-derived materials with polar and catalysis properties are more favorable for polysulfides confinement and transformation. The design and incorporation of multiple metal sites in MOFs or their derivatives can greatly improve catalytic activities toward ORR and OER, which are truly used as bifunctional catalyst Li–O_2_ batteries and Zn–air batteries. MOFs constructed with variable–valence metal center ions and redox-active organic ligands are beneficial for metal ions insertion in SIBs and PIBs, and ZIBs. For MOFs/MOF composite derivatives, integration of conductive carbon components and active metal-based species is an effective approach to prepare electrode materials for metal ion batteries. In addition, MOFs can protect the metal anode of Li- and Zn-based batteries. Both pristine MOFs with regular porous structure and MOF-based derivatives with lithiophilic active sites (e.g., N heteroatoms) can realize homogenous deposition and suppress dendrite formation of metal anode.

(4) Much more effort should be devoted to practical applications of MOF-based next-generation rechargeable batteries. Most of MOF-based energy materials can only be synthesized on the laboratory scale, which is complex and time-consuming. The development of facile and efficient approaches to the large-scale production of MOFs is still necessary. Moreover, the high cost and low yields of the MOF-based derivatives are disadvantageous to scalable production in practical applications. MOFs have shown great potential applications in new-generation batteries; however, the excellent electrochemical performance was mainly based on lab-scale (coin-type) cells. It is highly desirable to evaluate their real potentials in practical pouch cells. Although scientific researches and industrial applications of MOF-based batteries still faced challenges, it is undoubted that MOFs can serve as the high-capacity electrode materials, single ion conductor for solid-state electrolyte, or even protecting materials on metal anodes in next-generation batteries. In particular, some MOF-based materials can act as modified separators and functional interlayers to simultaneously inhibit the polysulfides and Li dendrites growth, largely facilitate the industrialization process of MOFs in Li–S battery applications [197, 198].

In conclusion, great progress of MOFs has been achieved in the field of next-generation batteries in recent years, the rational design of advanced MOF-based nanostructures/architectures is still in the early stages of research. Most of the studies are limited to the ZIFs family (ZIF-8 and ZIF-67) and PBAs. New strategies and preparation methods for synthesizing novel MOFs/MOF composites and their derivatives are more exploited for energy applications. Meanwhile, an in-depth investigation on fundamental mechanism in the electrochemical processes by in situ characterizations is highly required for future MOF-based material design and optimization. Moreover, the high cost and environmental damage of preparing MOFs still hinder their actual application, which needs further strenuous efforts in future researches. Although there are still several challenges, therefore, it is anticipated that the development of novel MOF chemistries and advanced technologies will provide numerous opportunities to achieve high-performance next-generation rechargeable batteries.
